# Supplementary material for: Engineered mRNA backbones for gene expression in human T cells
Source: Mol Ther Nucleic Acids. 2026 Mar 24;37(2):102913. doi: 10.1016/j.omtn.2026.102913 (PMC13101584; doi:10.1016/j.omtn.2026.102913)
Supplement: Document S1. Figures S1–S15 and Table S1 [file mmc1.pdf]

OMTN, Volume *37*

Supplemental information

Engineered mRNA backbones

for gene expression in human T cells

Gilad Gibor, Neve Tzvi, Amilia Meir, Hiba Abu-Hariri, Anat Shemer, Shai Kilim, Sophie Abelian, Ortal Harush, Orit Itzhaki, Ronnie Shapira-Frommer, Elad Jacoby, Gal Cafri, and Yochai Wolf

Supplemental Information

Table S1. All constructs used in the research

5'-UTR	*Accession number*	GOI
Globin (HBA1)	NM_00058	EGFP
IFN-gamma	NM_000619	EGFP
TIGIT	NM_173799	EGFP
LAG3	NM_002286	EGFP
TNF	NM_000594	EGFP
TOX	NM_014729	EGFP
GNZB	NM_004131	EGFP
IL-2	NM_000586	EGFP
CD3-epsilon	NM_000733	EGFP
CD3-zeta (CD247)	NM_000734	EGFP
CD39	NM_001776	EGFP
PD1	NM_005018	EGFP
TIM-3	NM_032782	EGFP
CD69	NM_001781	EGFP
TIGIT		Luc
Globin		Luc
IFN-gamma		Luc
TNF		Luc
LAG3		Luc
Globin		CD19-CAR
IFN-gamma		CD19-CAR
TNF		CD19-CAR
LAG3		CD19-CAR
TIGIT		CD19-CAR

A

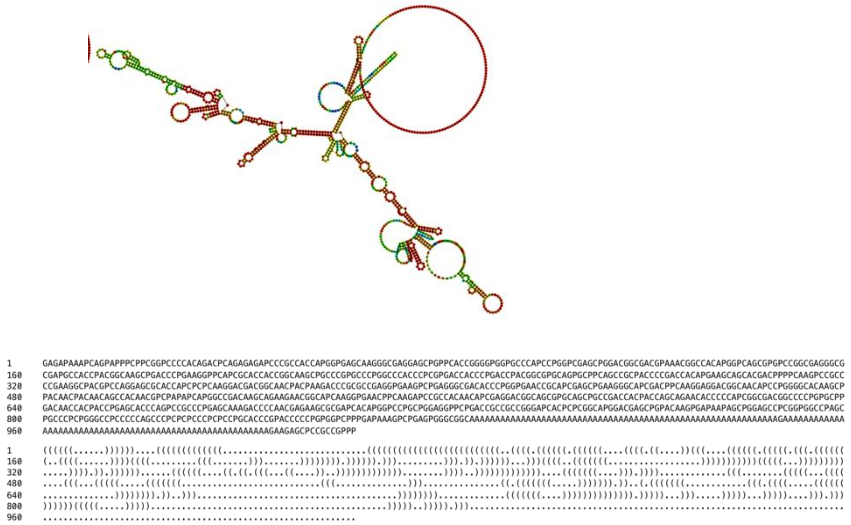

B

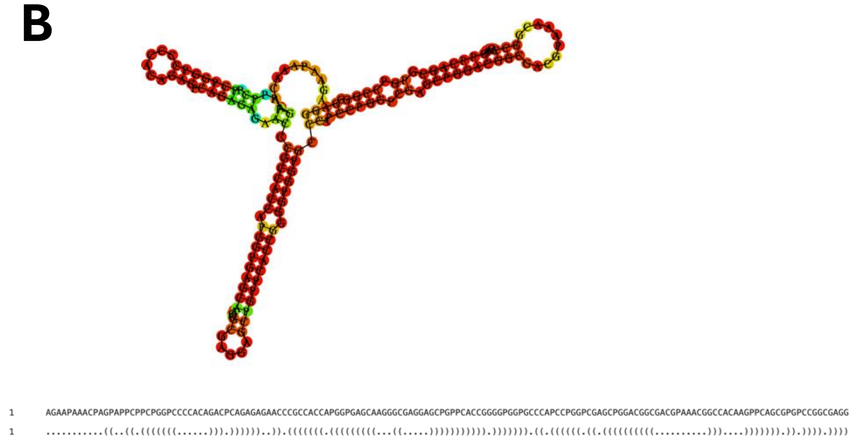

Figure S1 1. RNAFold analysis for 5'-HBA1-EGFP, both for its full structure (A) and its first 100 coding sequnce base pairs (B)

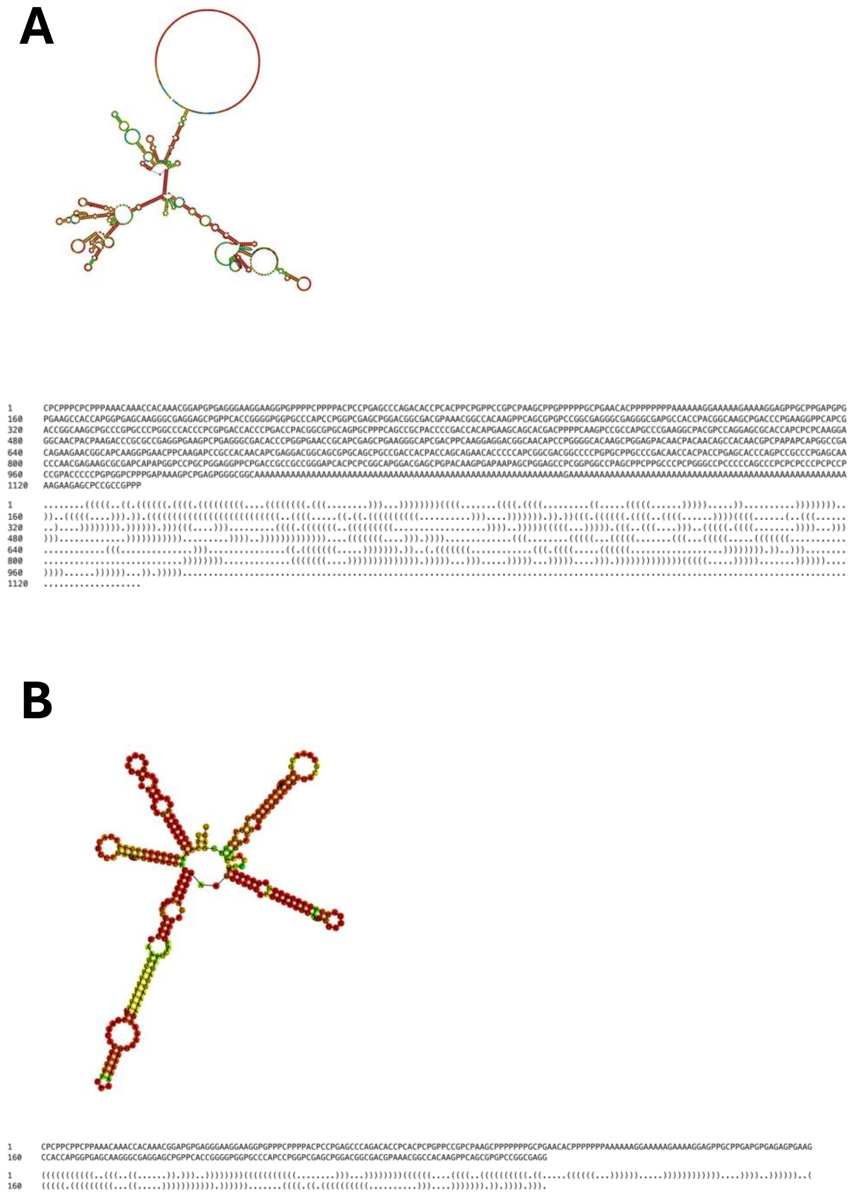

Figure S2. RNAFold analysis for 5'-TOX-EGFP, both for its full structure (A) and its first 100 coding sequnce base pairs (B)

A

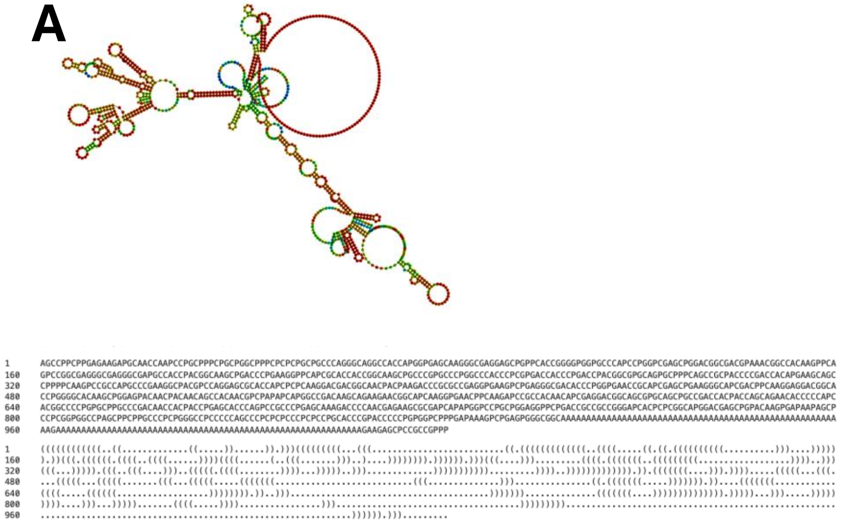

B

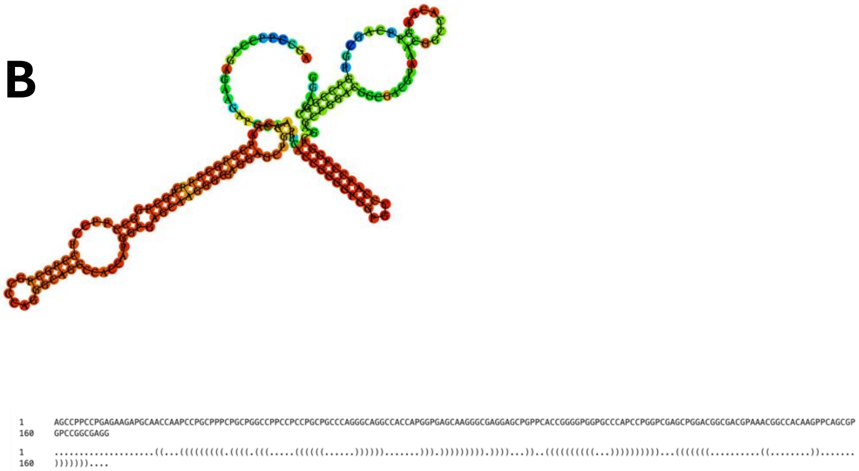

Figure S3. RNAFold analysis for 5’-GZMB-EGFP, both for its full structure (A) and its first 100 coding sequnce base pairs (B)

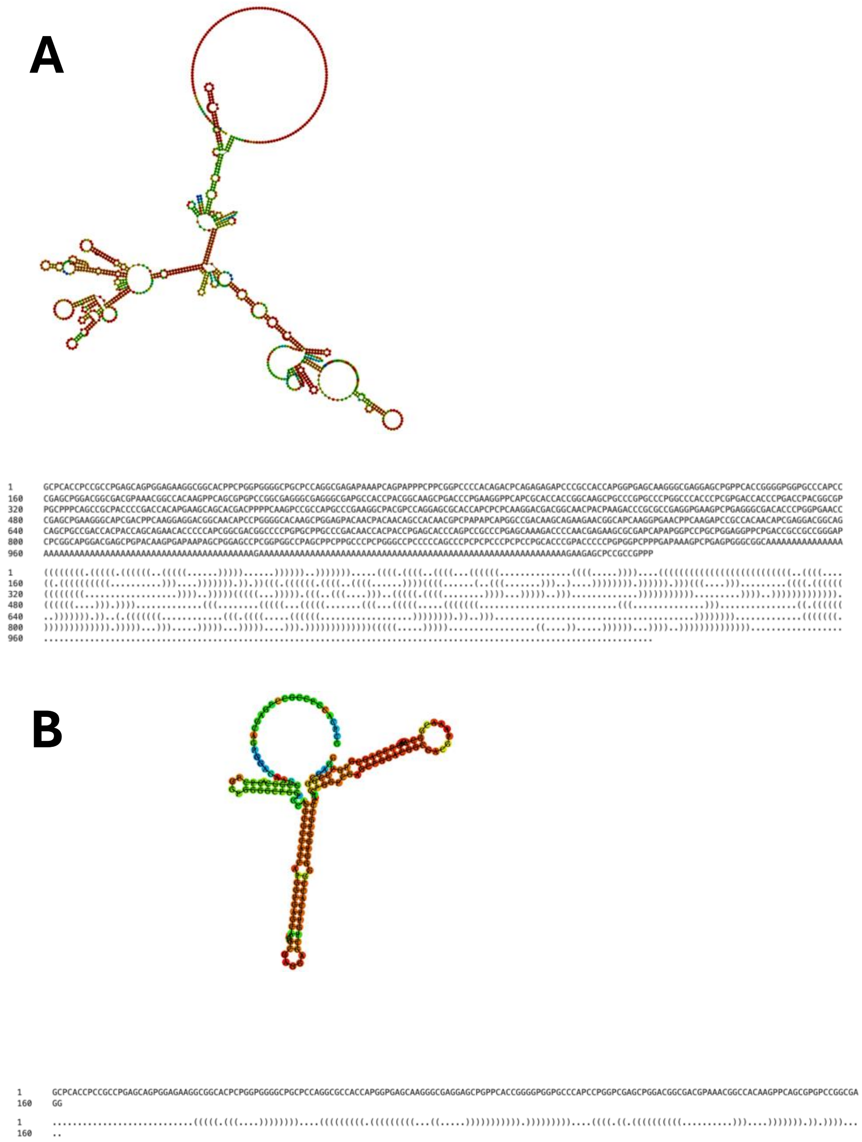

Figure S4. RNAFold analysis for 5'-PD1-EGFP, both for its full structure (A) and its first 100 coding sequnce base pairs (B)

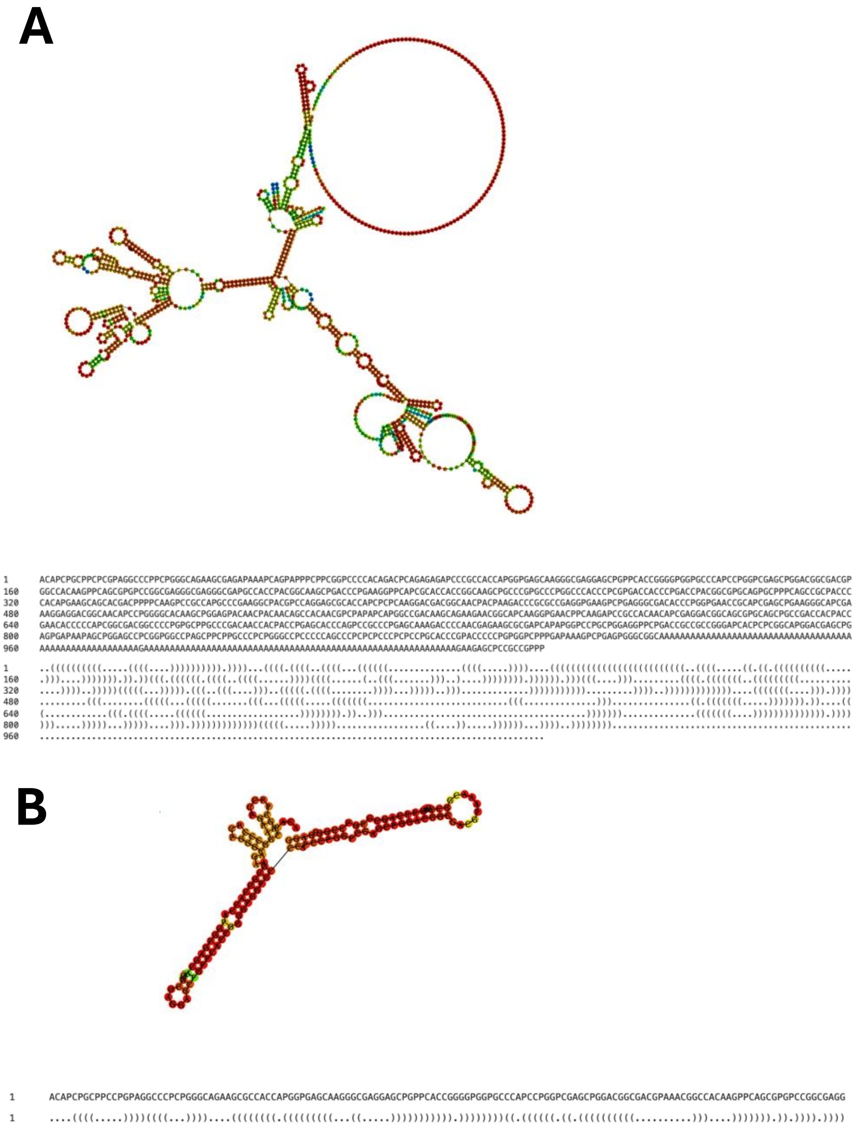

Figure S5. RNAFold analysis for 5'-TIGIT-EGFP, both for its full structure (A) and its first 100 coding sequnce base pairs (B)

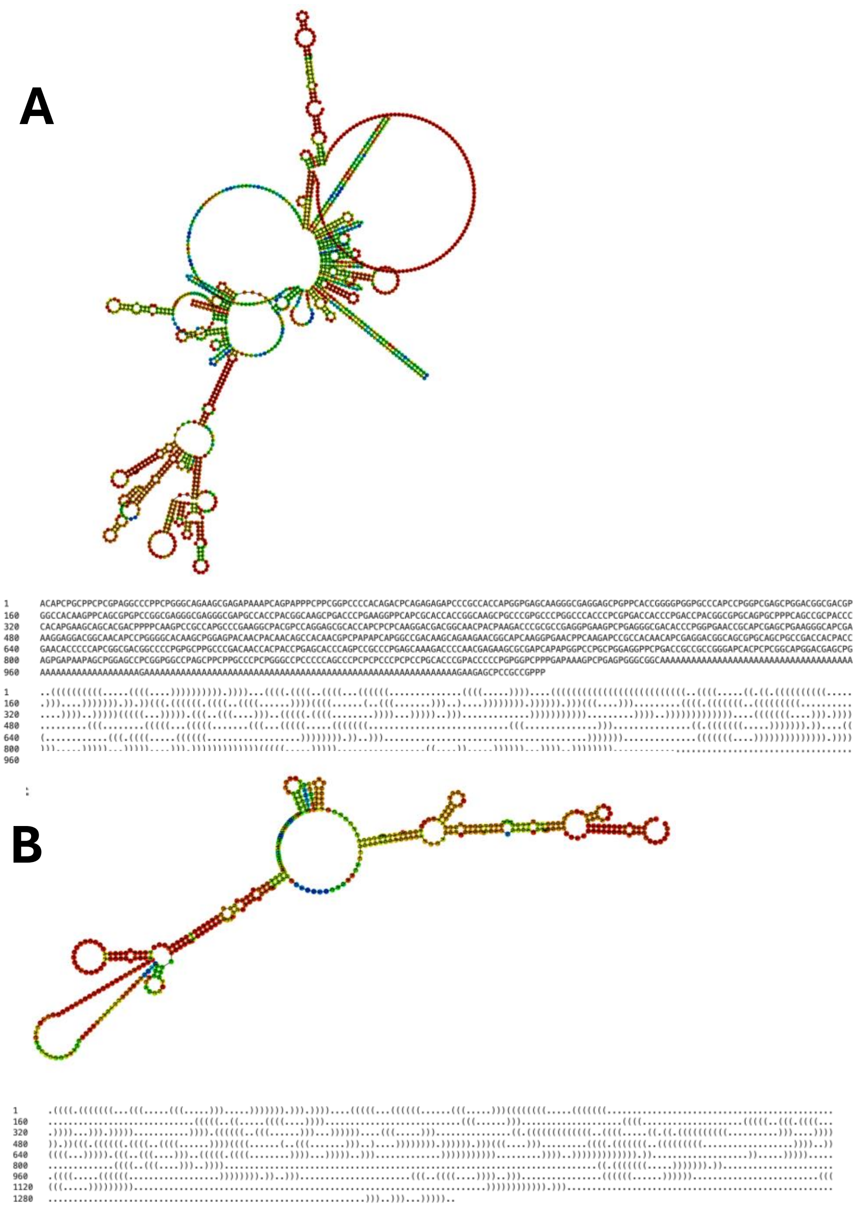

Figure S6. RNAFold analysis for 5'-LAG3-EGFP, both for its full structure (A) and its first 100 coding sequnce base pairs (B)

A

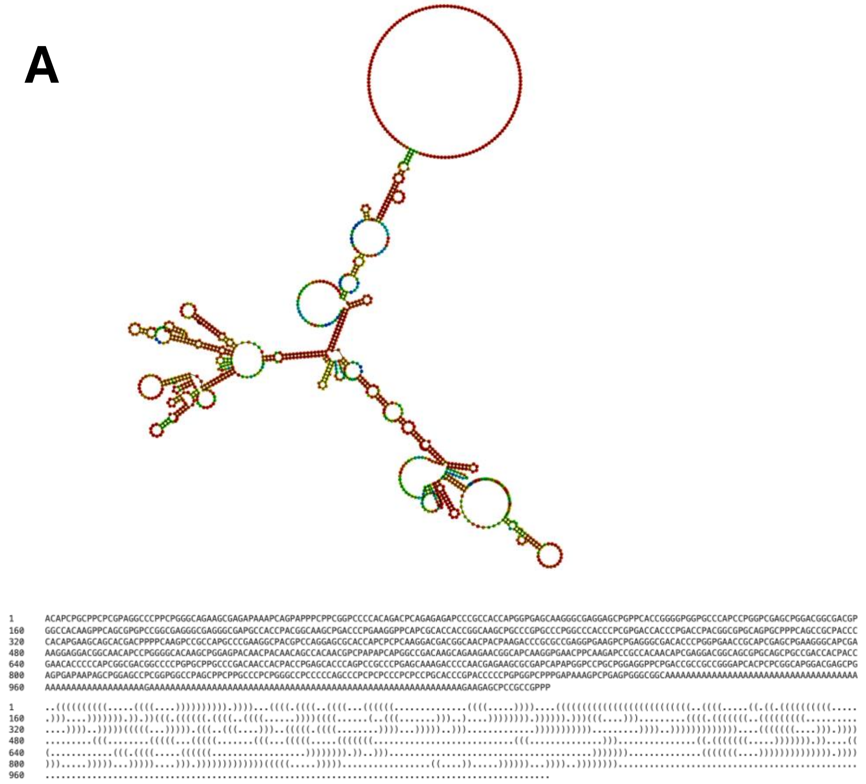

B

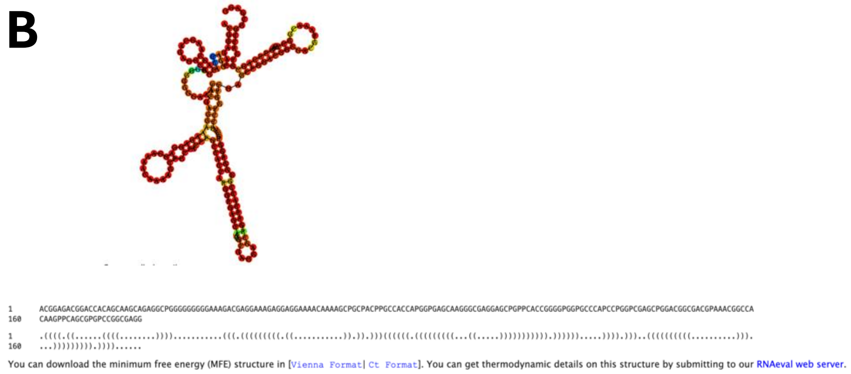

Figure S7. RNAFold analysis for 5'-CD39-EGFP, both for its full structure (A) and its first 100 coding sequnce base pairs (B)

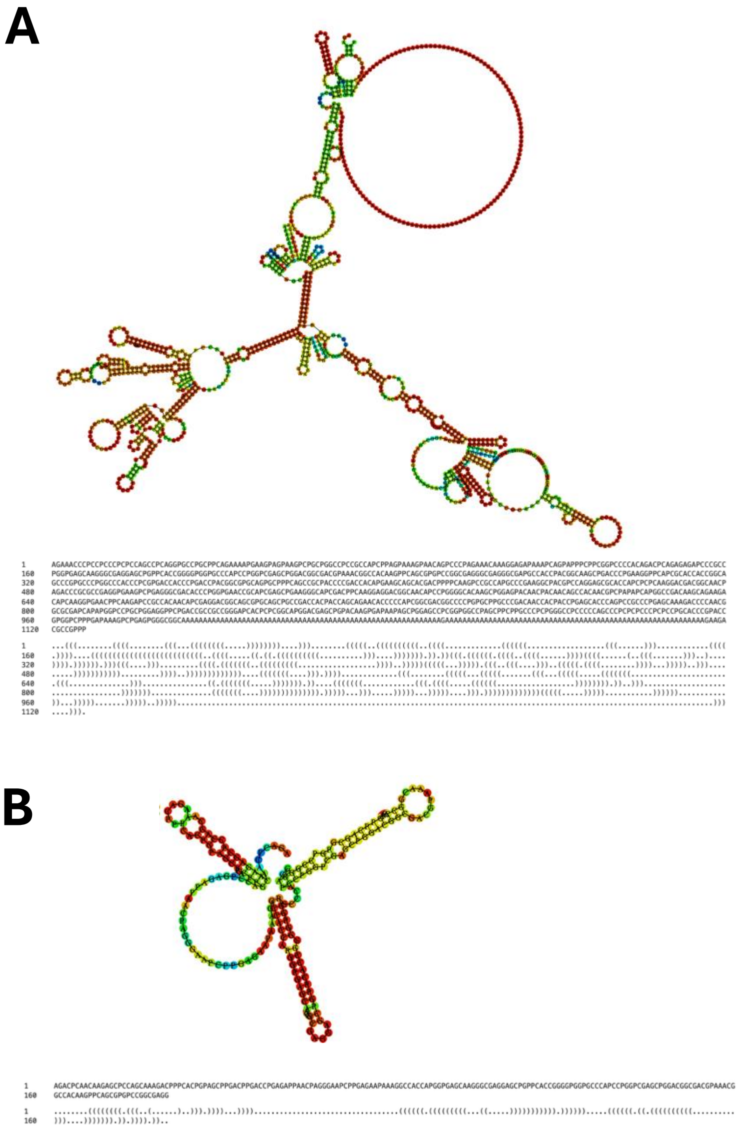

Figure S8. . RNAFold analysis for 5’-CD69-EGFP, both for its full structure (A) and its first 100 coding sequnce base pairs (B)

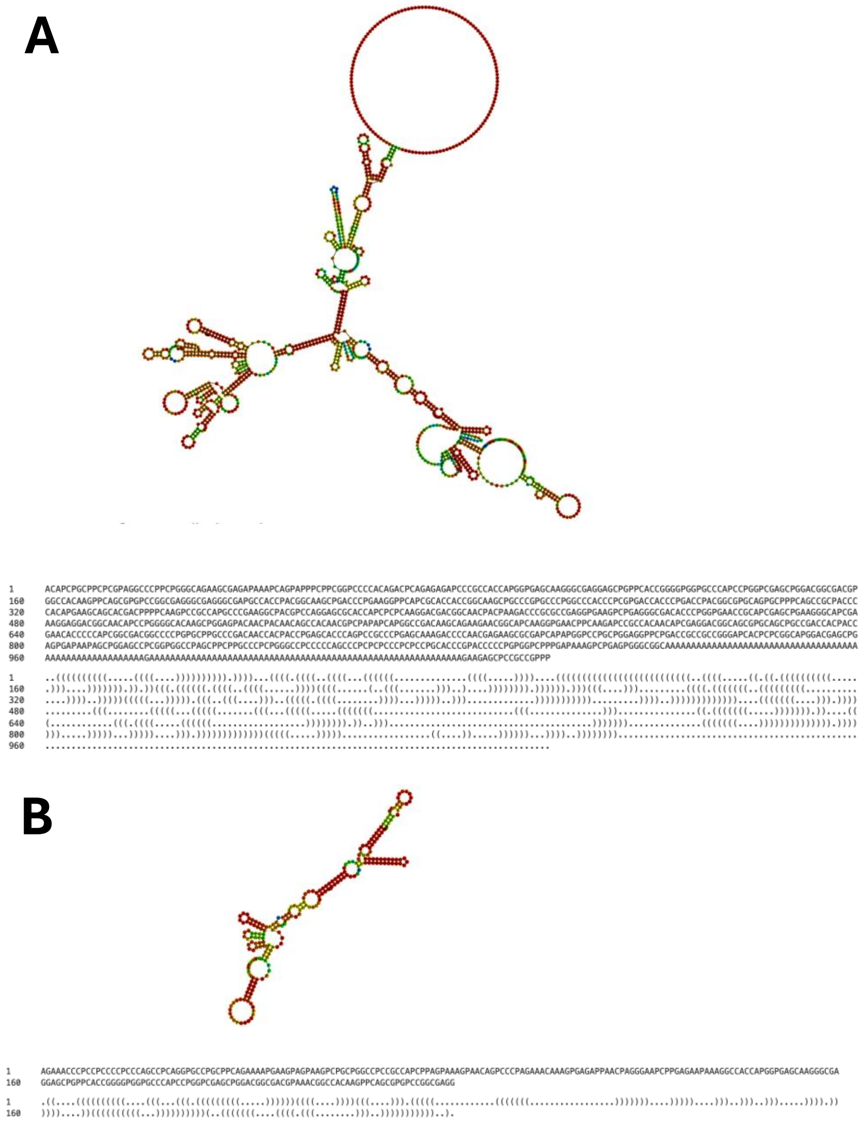

Figure S9. .RNAFold analysis for 5'-CD3e-EGFP, both for its full structure (A) and its first 100 coding sequnce base pairs (B)

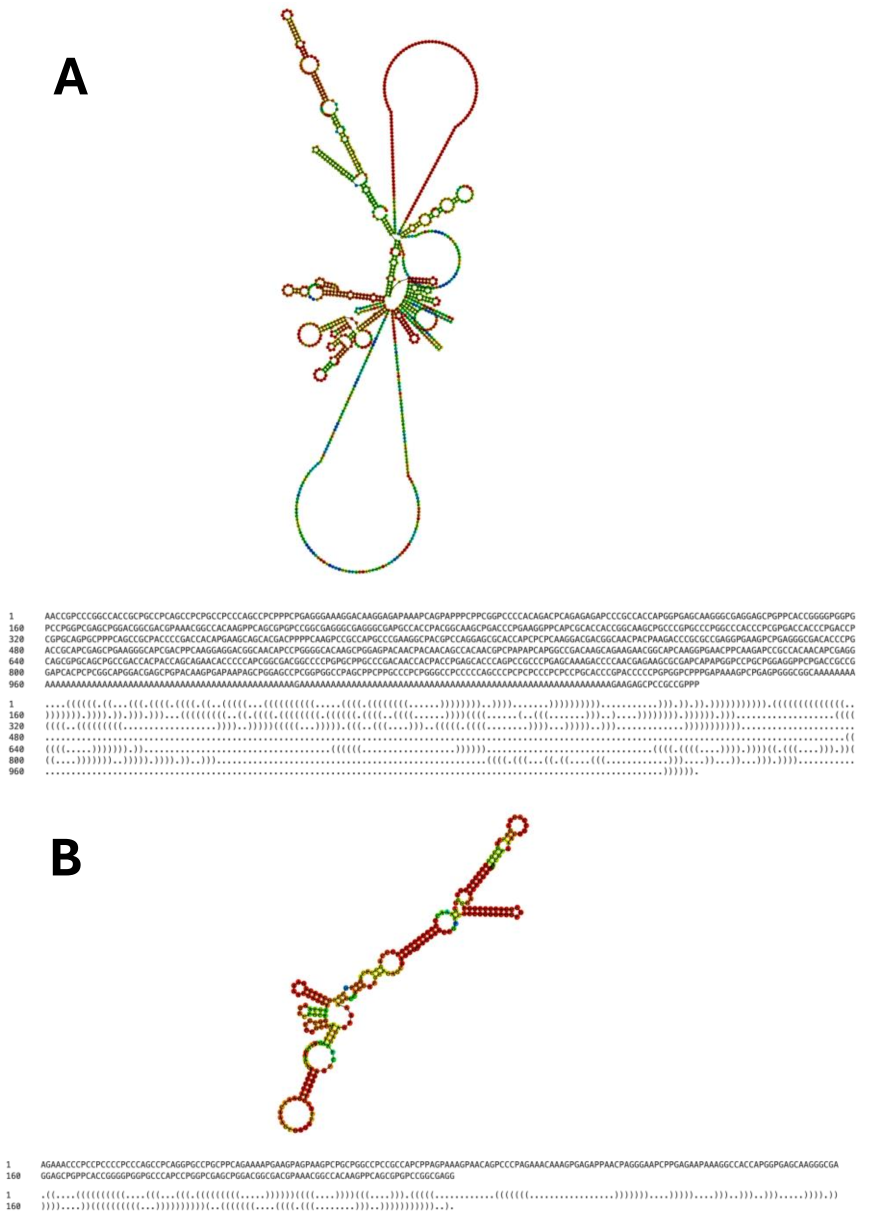

Figure S10. RNAFold analysis for 5'-CD247-EGFP, both for its full structure (A) and its first 100 coding sequnce base pairs (B)

A

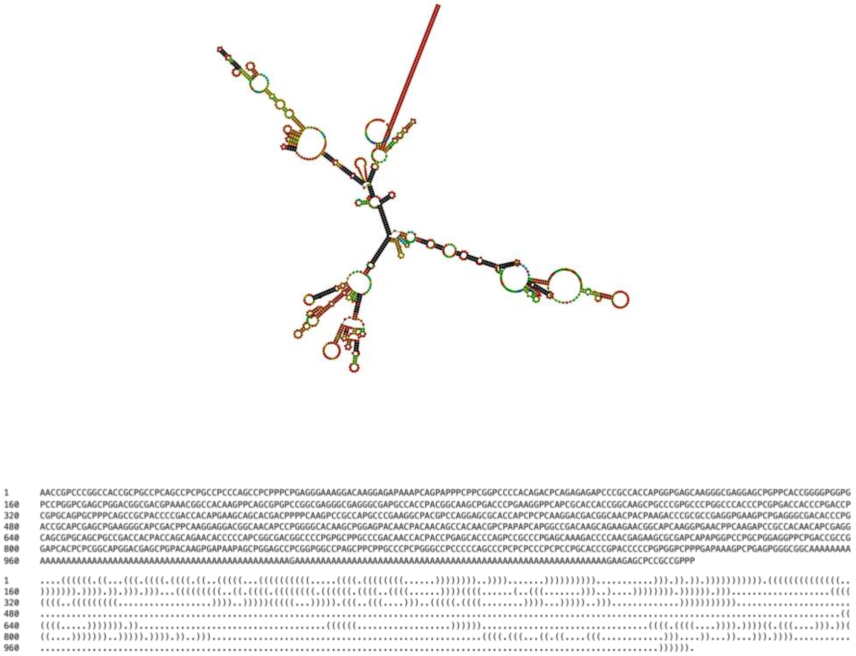

B

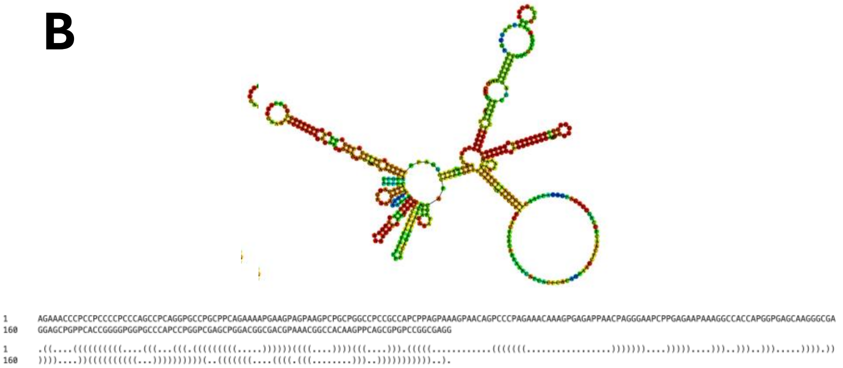

Il-2

Figure S11. RNAFold analysis for 5’-iL-2-EGFP, both for its full structure (A) and its first 100 coding sequnce base pairs (B)

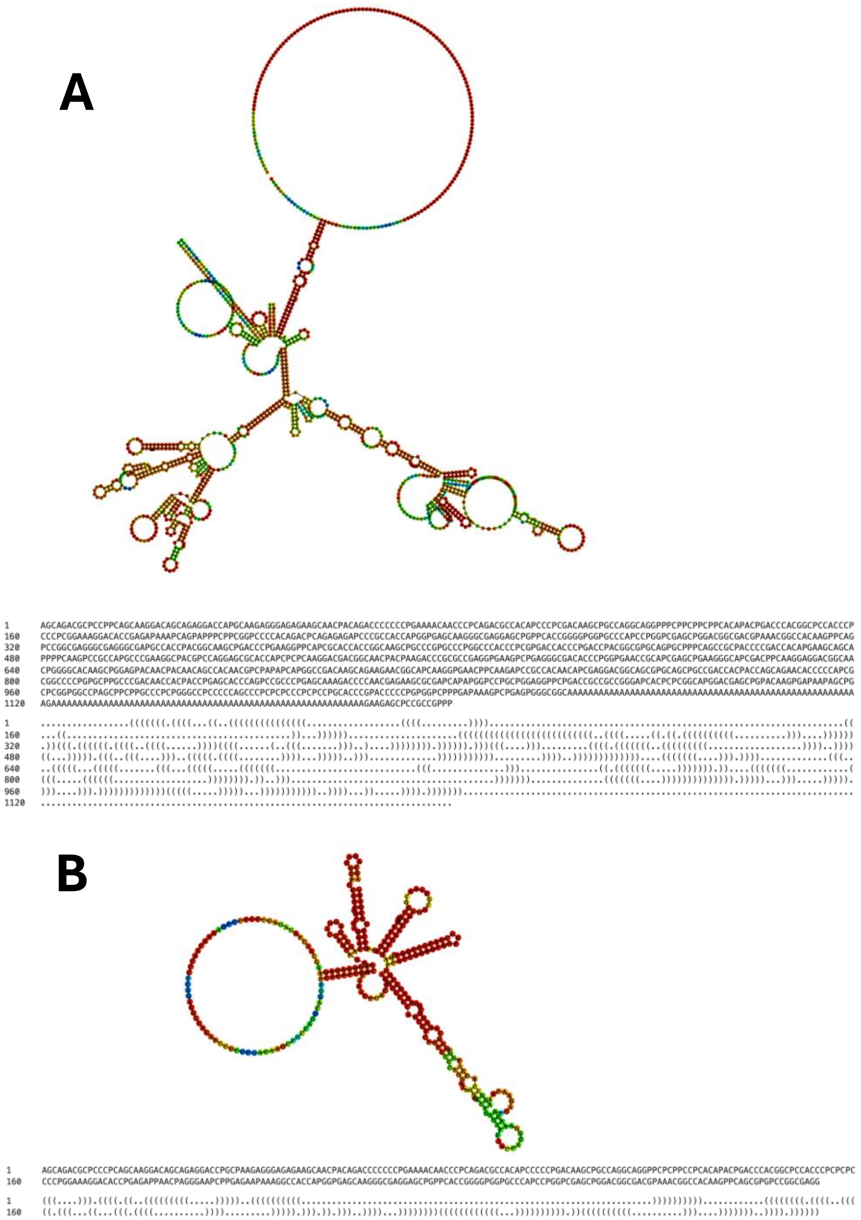

Figure S12. RNAFold analysis for 5'-TNF-EGFP, both for its full structure (A) and its first 100 coding sequnce base pairs (B)

A

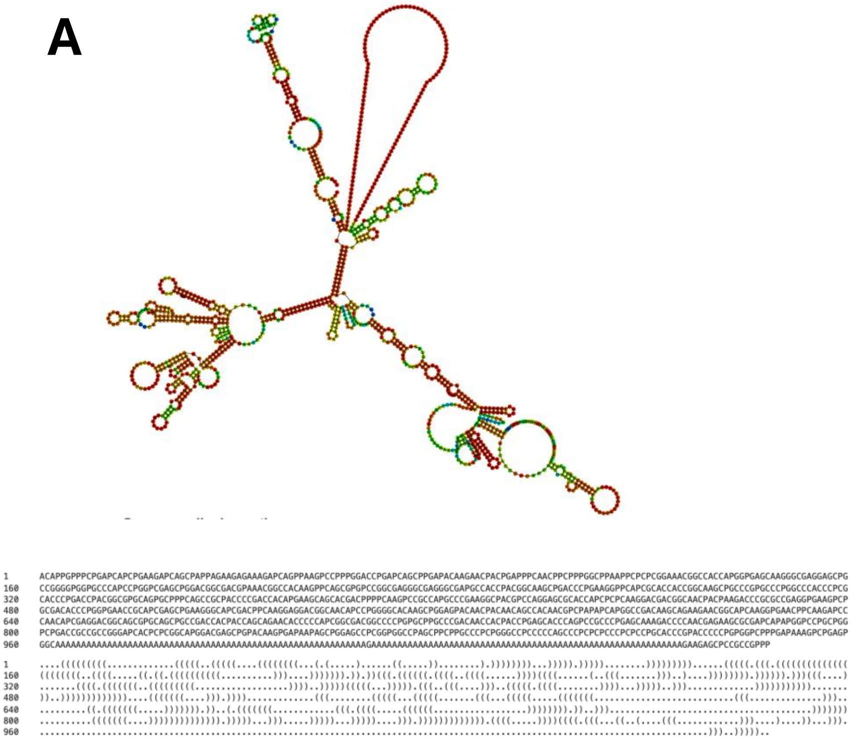

B

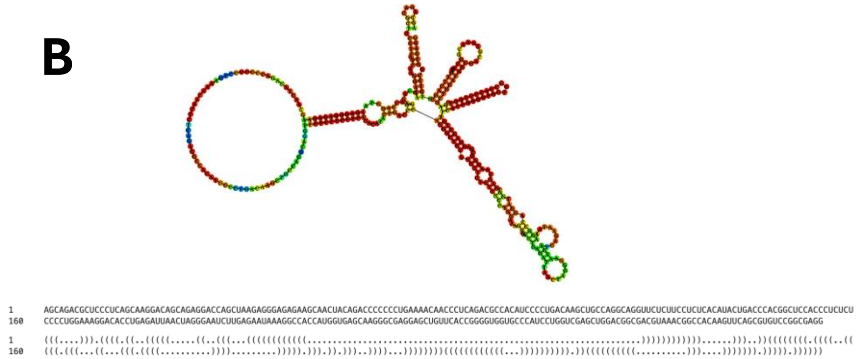

Figure S13. RNAFold analysis for 5'-IFNG-EGFP, both for its full structure (A) and its first 100 coding sequnce base pairs (B)

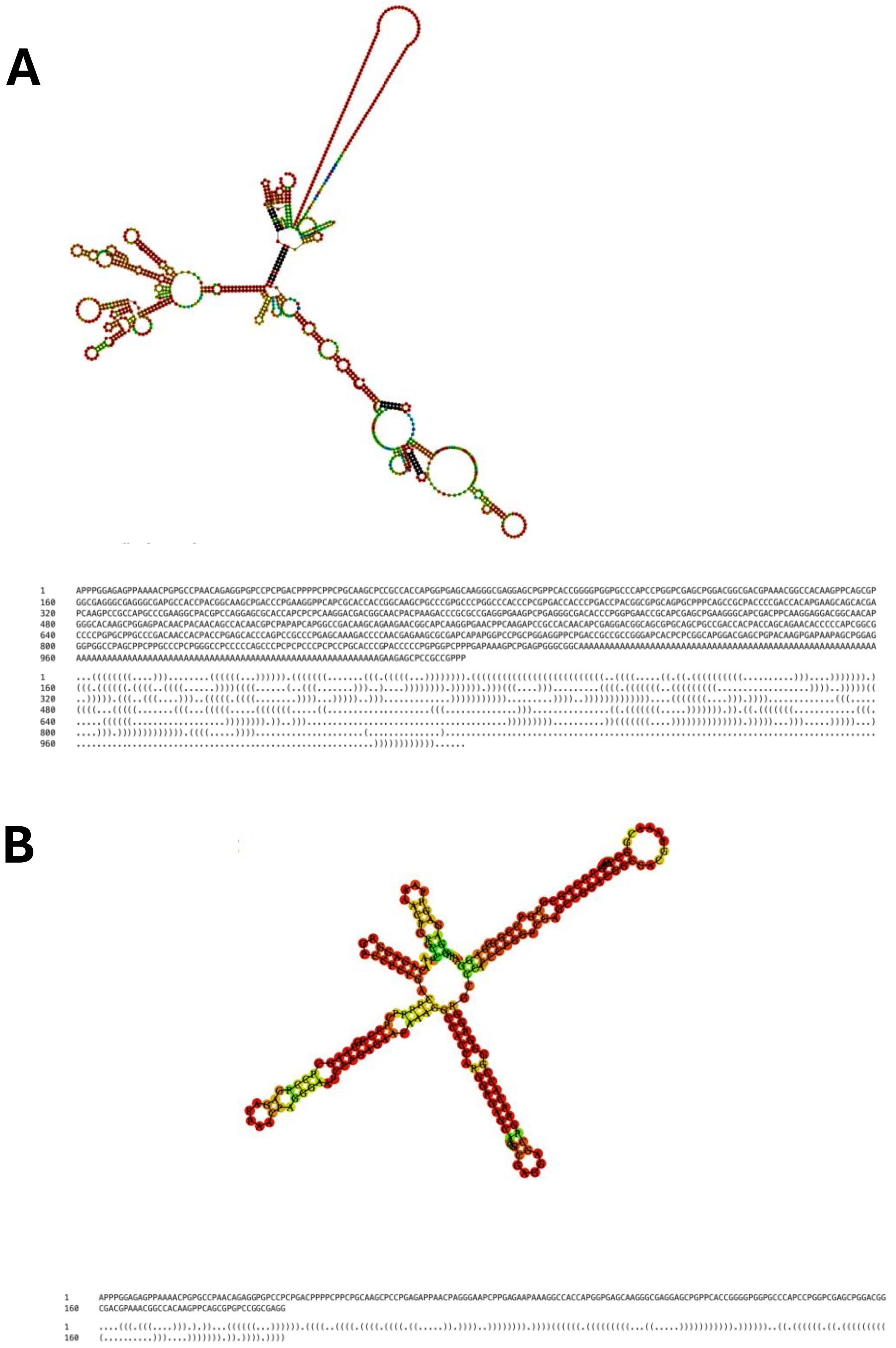

Figure S14. RNAFold analysis for 5'-TIM3F-EGFP, both for its full structure (A) and its first 100 coding sequnce base pairs (B)

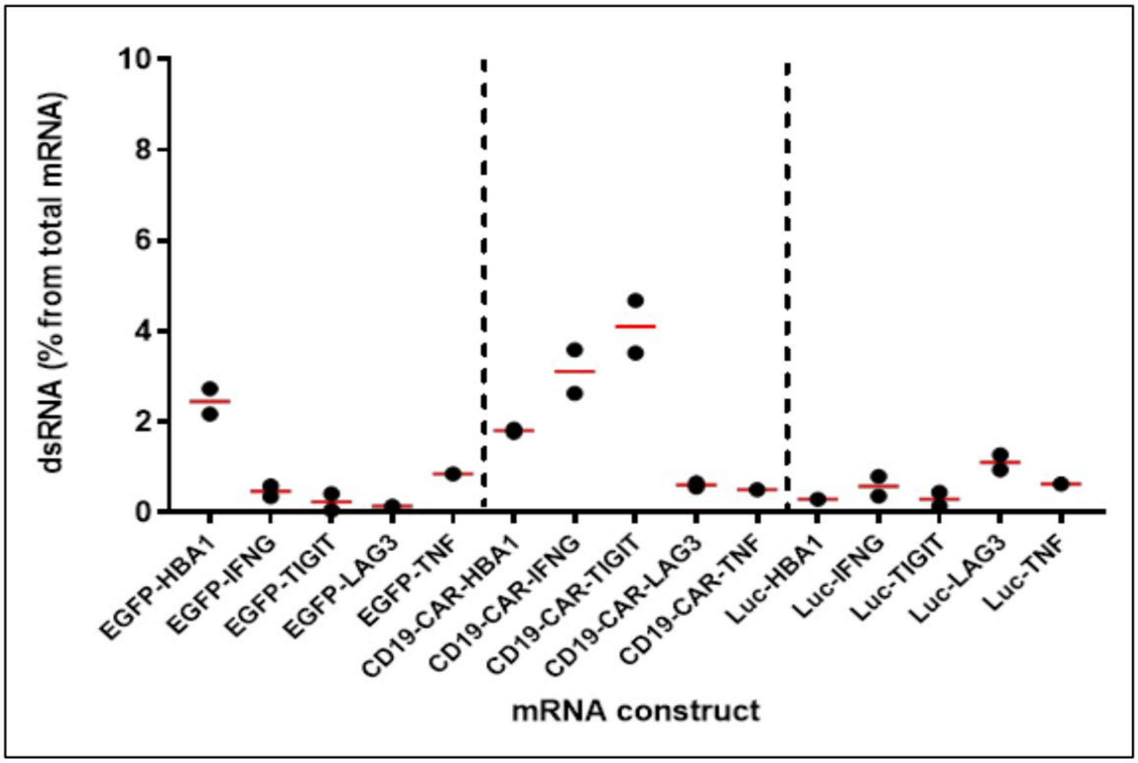

Figure S15. dsRNA content in IVT mRNA preparations. dsRNA was quantified using a J2-based anti-dsRNA assay (J2 dsRNA ELISA; Biorbyt) and expressed as a percentage of total mRNA Input for each construct. Points represent independent technical repeats; red bars indicate the mean. Values were interpolated from a positive-control standard curve using a sigmoidal 4-parameter logistic (4PL) fit
