# Supplementary material for: Engineered mRNA backbones for gene expression in human T cells
Source: Mol Ther Nucleic Acids. 2026 Mar 24;37(2):102913. doi: 10.1016/j.omtn.2026.102913 (PMC13101584; doi:10.1016/j.omtn.2026.102913)
Supplement: Document S2. Article plus supplemental information [file mmc2.pdf]

Molecular Therapy
Nucleic Acids
Brief report

Engineered mRNA backbones for gene expression in human T cells

Gilad Gibor,[1] Neve Tzvi,[1] Amilia Meir,[2] Hiba Abu-Hariri,[3] Anat Shemer,[3] Shai Kilim,[1] Sophie Abelian,[4] Ortal Harush,[1] Orit Itzhaki,[1] Ronnie Shapira-Frommer,[1] Elad Jacoby,[2,5,6] Gal Cafri,[3,7,9] and Yochai Wolf[1,8,9]

[1]Ella Lemelbaum Institute for Immuno-oncology, Sheba Medical Center, Tel Hashomer, Ramat Gan, Israel; [2]Cell Therapy Lab, Sheba Medical Center, Tel Hashomer, Ramat Gan, Israel; [3]Immunotherapy and Genetic Engineering, Sheba Medical Center, Tel Hashomer, Ramat Gan, Israel; [4]University Collage London, London, UK; [5]Gray Faculty of Medical & Health Sciences, Tel Aviv University, Tel Aviv, Israel; [6]Division of Pediatric Hematology and Oncology, The Edmond and Lily Safra Children's Hospital, Tel Hashomer, Ramat Gan, Israel; [7]Dina Recanati School of Medicine, Reichman University, Herzliya, Israel; [8]Department of Pathology, Gray Faculty of Medicine, Tel Aviv University, Tel Aviv, Israel

Current mRNA approaches in immuno-oncology lack specificity for optimal T cell mRNA expression, necessitating tailored mRNA expression systems. In this study, we developed novel mRNA constructs in which the standard α-globin (HBA1) 5′ UTR is replaced with sequences derived from genes highly expressed in effector T cells. Using primary human T cells, expression levels of UTR-modified reporter genes were evaluated, revealing significant variability based on the substituted UTR. For instance, interferon gamma (IFN-γ) UTRs facilitated enhanced and sustained protein expression, whereas TNF UTRs showed diminished expression. Unexpectedly, the *in silico*-predicted RNA stability of the various UTR-modified constructs did not correlate with the altered expression. These UTR-mediated differences in protein expression were unique to T cells, as HEK cells introduced with the same constructs showed distinct expression profiles. CD19-CAR constructs expressed in T cells using various 5′ UTRs demonstrated different protein expression and function toward antigen-positive target cells, as well as tonic signaling, manifested by the immune output in the absence of antigen. Specifically, for CD19-CAR, using the TIGIT 5′ UTR proved optimal for achieving maximal reactivity while minimizing tonic signaling. These findings provide proof of concept for the pivotal role of T cell-specific UTRs in optimizing CAR-T cell functionality by fine-tuning expression, reducing tonic signaling, and minimizing off-target effects, thus emphasizing their potential in advancing the therapeutic potential of mRNA-based CAR-T cell therapies.

INTRODUCTION

mRNA is a powerful gene expression platform for vaccines, cell engineering, and regenerative medicine, offering advantages over DNA transfection and viral methods. It is considered safe due to its lack of genomic integration and off-target effects, while enabling high expression of multiple genes for cellular reprogramming.[1–3] The function of mRNA depends on its half-life, bioavailability, and translation efficiency, which are influenced by its 5′ and 3′ untranslated regions (UTRs). α-Globin (*HBA1*)-derived UTRs, commonly used in mRNA therapeutics, enhance stability but are not optimized for T cell types. Previous attempts to modify either 3′- or 5′ UTR in cell types other than T cells proved successful in fine-tuning and optimizing cell-specific gene expression,[4,5] particularly the 5′ UTR.[6]

mRNA-based CAR-T cells represent an innovative and promising strategy in cancer immunotherapy, offering a flexible and potentially safer alternative to traditional methods such as viral-based CAR-T therapies. mRNA CAR-T cells lead to transient CAR expression, which can reduce risks associated with CAR off-target effects and CAR-T cell exhaustion. Additionally, mRNA-based CAR-T cells can be re-dosed, allowing for easier control over therapeutic levels, which may enhance safety and enable fine-tuning of the immune response over time. Ultimately, mRNA-CARs may provide a safer, cost-effective, and rapidly deployable approach for cancer immunotherapy, potentially allowing direct *in vivo* CAR expression through lipid nanoparticle (LNPs), as seen in mRNA-based vaccines.[7,8]

Here, to enhance mRNA expression in activated human T cells, we investigate 5′ UTRs linked to T cell activation and function. We demonstrate the effects of various 5′ UTRs by replacing the *HBA1*-derived UTR, utilizing reporter genes such as GFP and luciferase, and using CD19-CAR as a functional example. This approach aims to optimize mRNA expression *in vivo* for therapeutic applications.

RESULTS

The molecular sequence of each mRNA used in our study included the 5′ anti-reverse cap analog (ARCA), 5′ UTR, open reading frame

Received 15 August 2025; accepted 20 March 2026;
https://doi.org/10.1016/j.omtn.2026.102913.

[9]These authors contributed equally

Correspondence: Gal Cafri, Immunotherapy and Genetic Engineering, Sheba Medical Center, Tel Hashomer, Ramat Gan, Israel.
E-mail: gal.cafri@sheba.health.gov.il
Correspondence: Yochai Wolf, Ella Lemelbaum Institute for Immuno-oncology, Sheba Medical Center, Tel Hashomer, Ramat Gan, Israel.
E-mail: yochai.wolf@sheba.health.gov.il

© 2026 The Author(s).
Published by Elsevier Inc. on behalf of The American Society of Gene and Cell Therapy.
This is an open access article under the CC BY license (http://creativecommons.org/licenses/by/4.0/).

(ORF), 3′ UTR, and 3′ poly(A) tail composed of 120 A. All mRNAs were synthesized with pseudouridine-5′-triphosphate (pseudo-UTP). We then resorted to screening T cell activation/exhaustion-associated UTRs. As proof of concept, we replaced the 5′ UTR of HBA1 with the new 5′ UTRs to achieve compatibility with T cell expression. We designed a library of 13 mRNA constructs with 5′ UTR derived from proteins highly and specifically expressed in activated or exhausted T cells, such as cytokines (*IL2*, *IFNG*, and *TNF*), effector molecules (granzyme B [*GZMB]* and CD39 [*ENTPD1]*), and checkpoint molecules (PD1 *[PDCD1*] and TIM3 *[HAVCR2]*) (Figure 1A; see Table S1 for all constructs used in the study). We first designed constructs encoding for *EGFP*, and retained the 3′ *HBA1* UTR, mainly due to the longer length of the 3′ UTRs (ranging from 100 bp to several thousands) compared to 5′ UTRs (50–300 bp).[9] To evaluate whether simple compositional features could predict the expression patterns of the tested UTRs, we analyzed their length, GC/AU content, and nucleotide distribution (Figure 1B). The 5′ UTR of cytokine IL-2 had the lowest G/C content (35%) and highest A/U content, which could predict minimal secondary structure and optimal translation initiation, whereas the 5′ UTR of *TNF* had the highest C/G (74%) and lowest A/U, indicating possible high frequency of secondary structures which may form long helices and internal dsRNA segments, and thus may activate the dsRNA sensor protein kinase R (PKR).[10] In terms of length, the 5′ UTR of *TIGIT* is the shortest (34 bp), whereas the 5′ UTR of *LAG3* is the longest (333 bp). To predict which mRNA construct would enhance or diminish expression, we used the RNAfold online algorithm (http://rna.tbi.univie.ac.at/cgi-bin/RNAWebSuite/RNAfold.cgi),[11] which enables predictions of minimal free energy for each construct. For instance, while the centroid (the most representative structure of all possible folds) free energy, for the *HBA1* 5′-UTR-*EGFP* was $\Delta G = -304.6$, TOX 5′-UTR-EGFP was predicted to have the lowest centroid energy and thus the most stable, CD39 5′-UTR-EGFP was predicted to have the highest energy and thus the least stable (Figure 1C). Since we intended for mRNA synthesis to use ∼70% pseudouridine, which enhances local base-pair stability while disrupting the extended A-form duplexes required for PKR activation, resulting in more stable and translationally efficient mRNA with reduced innate immune sensing compared to unmodified uridine,[12–14] we recalculated the centroid free energy for each construct, considering energy correction for pseudouridine. This analysis showed different analysis, which recognized IL-2 5′ UTR as the construct predicted to be the most stable, and *CD247* (CD3 ζ) as the least stable (Figure 1D). As expected, the average centroid free energy for all 14 constructs was reduced by $\Delta G = -26.65$ ($p < 0.05$, paired t test), indicating that the usage of pseudouridine is expected to minimize dsRNA contamination and PKR activation (Figure 1E). According to the pseudouridine-corrected, predicted free energy re-calculation, 10 constructs were predicted to be more stable than *HBA1* 5′-UTR-*EGFP*, while 3 were predicted to be less stable (Figure 1F). Importantly, in all constructs, the longest RNA helix was 8–12 bp; no region contained ≥20 consecutive base pairs. Thus, the predicted structures do not form long dsRNA helices of the type known to activate PKR, as none of the 14 constructs reach the ≥20–30 bp threshold required for PKR activation.[15,16] To demonstrate the power of this platform, we initiated a screening process utilizing candidate 5′ UTRs for mRNA encoding EGFP and luciferase. Constructs were electroporated into human blood-derived T cells and expression was evaluated by flow cytometry following electroporation. We observed increased expression of reporter genes with several 5′ UTRs, such as the 5′ UTR of *IFNG*, and reduced protein expression with others, such as the 5′ UTR of *TNF* (Figures 2A and 2B). Strikingly, previous RNA stability prediction shown in Figure 1 was uncorrelated with actual expression; for instance, *IL2* 5′ UTR did not enhance EGFP expression compared to *HBA1* (Figure 2C). G/C content could also poorly predict actual expression. In contrast, the 5′ UTR of *TNF* and *PDCD1* did have low expression compatible with its high G/C content; other 5′ UTR with such high content, such as *TIGIT* and *LAG3*, had higher expression than that of *HBA1*, and *IL2* 5′ UTR, which had both the lowest GC content and the lowest predicted free energy, did not perform better than HBA1. Following our initial screen, we chose to work with the 5′ UTRs of I*FNG, LAG3*, and *TIGIT* as prime candidates of UTRs that enhance expression, and TNF, which consistently decreased GFP expression. We next confirmed the expression using luciferase-expressing mRNA in blood-derived T cells and melanoma-derived in-house tumor-infiltrating lymphocytes (TILs). In both, we confirmed that the *IFNG* 5′ UTR elevated luciferase activity almost 2-fold, while the *TNF* 5′ UTR decreased it by nearly 50% (Figures 2D and 2E). The tunable expression of mRNA constructs by these 5′ UTRs was T cell exclusive, as was electroporation of the constructs encoding for GFP (Figures 2F and 2G) or luciferase (Figure 2H) into HEK cells did not recapitulate the expression patterns observed in T cells. Furthermore, dsRNA levels were experimentally measured for all mRNA constructs used in the study and were generally low, indicating that the dsRNA burden does not support a uniform dsRNA-driven explanation for the observed functional differences (Figure S15).

Following the proof of concept of differential reporter gene expression using T cell-tailored UTRs, we incorporated these 5′ UTRs in mRNA constructs that encode for the CD19-CAR, allowing for both cytometric and functional validation of the expression. We then designed constructs encoding for CD19-CAR, which utilize the same 5′ UTRs used for reporter genes in Figure 2. For this purpose, we used an FMC63-CD28-CD3ζ CAR, as routinely manufactured in our institution,[17] and incorporated *TIGIT, IFNG, LAG3*, and *TNF* 5′ UTRs. We saw confirmed differential expression of the CD19-CAR driven by different 5′ UTRs. At the same time, the percentages of CD19-CAR^{+} cells vary with a decrease in TNF 5′ UTR but are not statistically significant (Figures 3A and 3B). The mean fluorescent intensity (MFI) of the constructs is statistically different, with *TNF*-5′ UTR being the least expressed (Figure 3C). This finding was consistent between CD19-CAR, GFP, and luciferase in T cells. It demonstrated that the level of CAR expression, rather than the penetrance of the construct, is more affected due to the 5′ UTR replacement. Interestingly, unlike constructs encoding for GFP and luciferase, the *IFNG* 5′ UTR showed no advantage

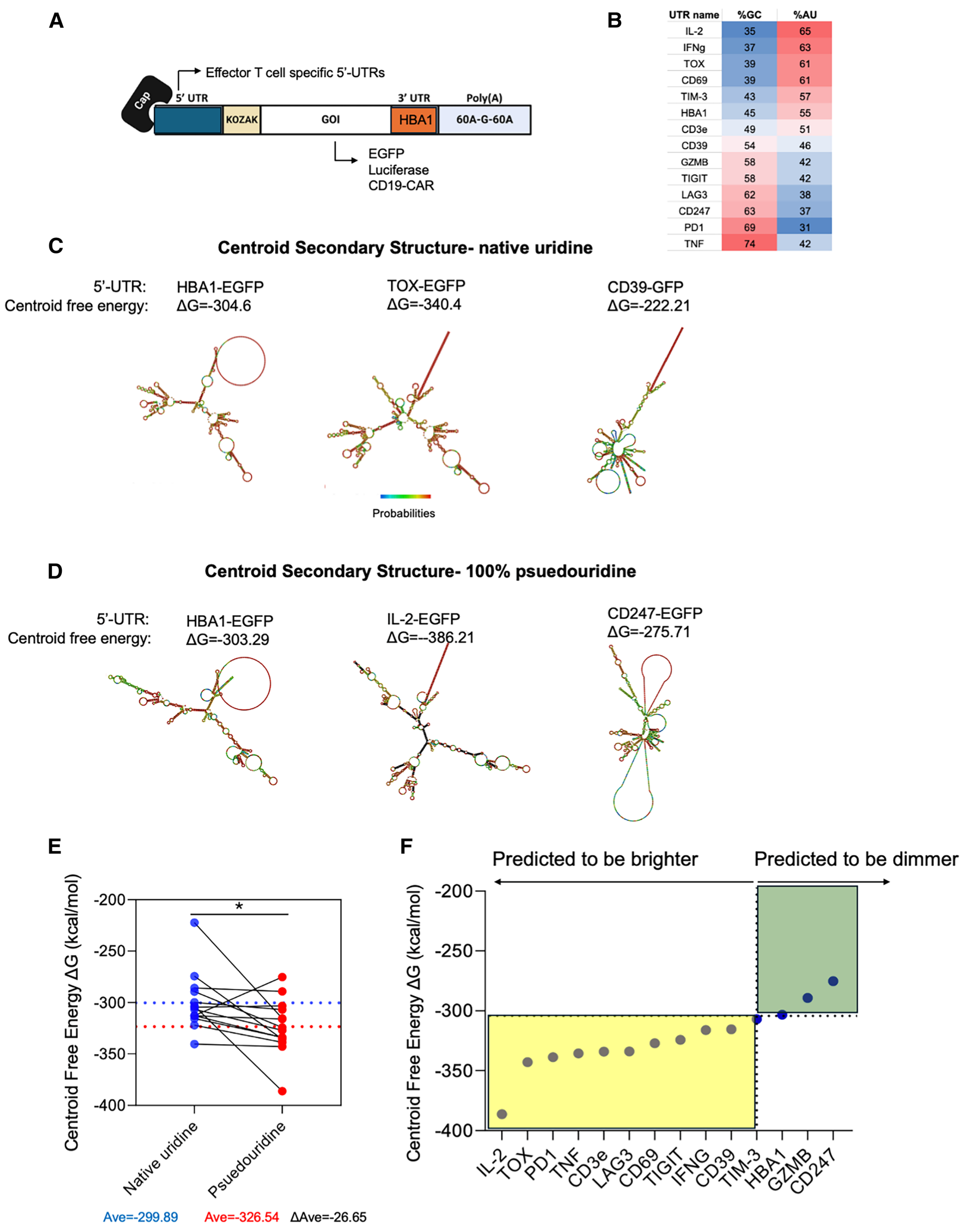

Figure 1. Generation of 5′ UTR library for T cell-specific gene expression

(A) General scheme of mRNA constructs used throughout the research. The constructs are composed of replaceable 5′ UTRs, KOZAK sequence, a gene of interest (GOI), a conserved 3′ UTR of HBA1 which is shared in all constructs, and a poly-A sequence. (B) GC/AU contents for each 5′ UTR used in the study. (C) Centroid secondary structure and free energy for EGFP-encoding constructs using the 5′ UTR of *TOX*, *HBA1*, and CD39 (*ENTPD1*). (D) Psuedouridine-corrected centroid secondary structure and free energy for EGFP-encoding constructs using the 5′ UTR of *IL2*, *HBA1*, and *CD247*. (E) Centroid free-energy calculation for all 14 constructs using either native uridine or pseudouridine. The blue-dashed line represents the mean of the native uridine calculation, and the red line represents the mean of the psuedouridine calculation. *$p < 0.05$, paired *t* test. (F) Distribution of centroid free energy of all 14 *EGFP* encoding constructs including *HBA1* 5′ UTR, corrected for the usage of pseudouridine instead of native uridine. Constructs with energy lower than *HBA1* 5′ UTR are predicted to be more stable and thus enhance expression, while constructs with energy higher than *HBA1* 5′ UTR are predicated to be less stable and impair expression.

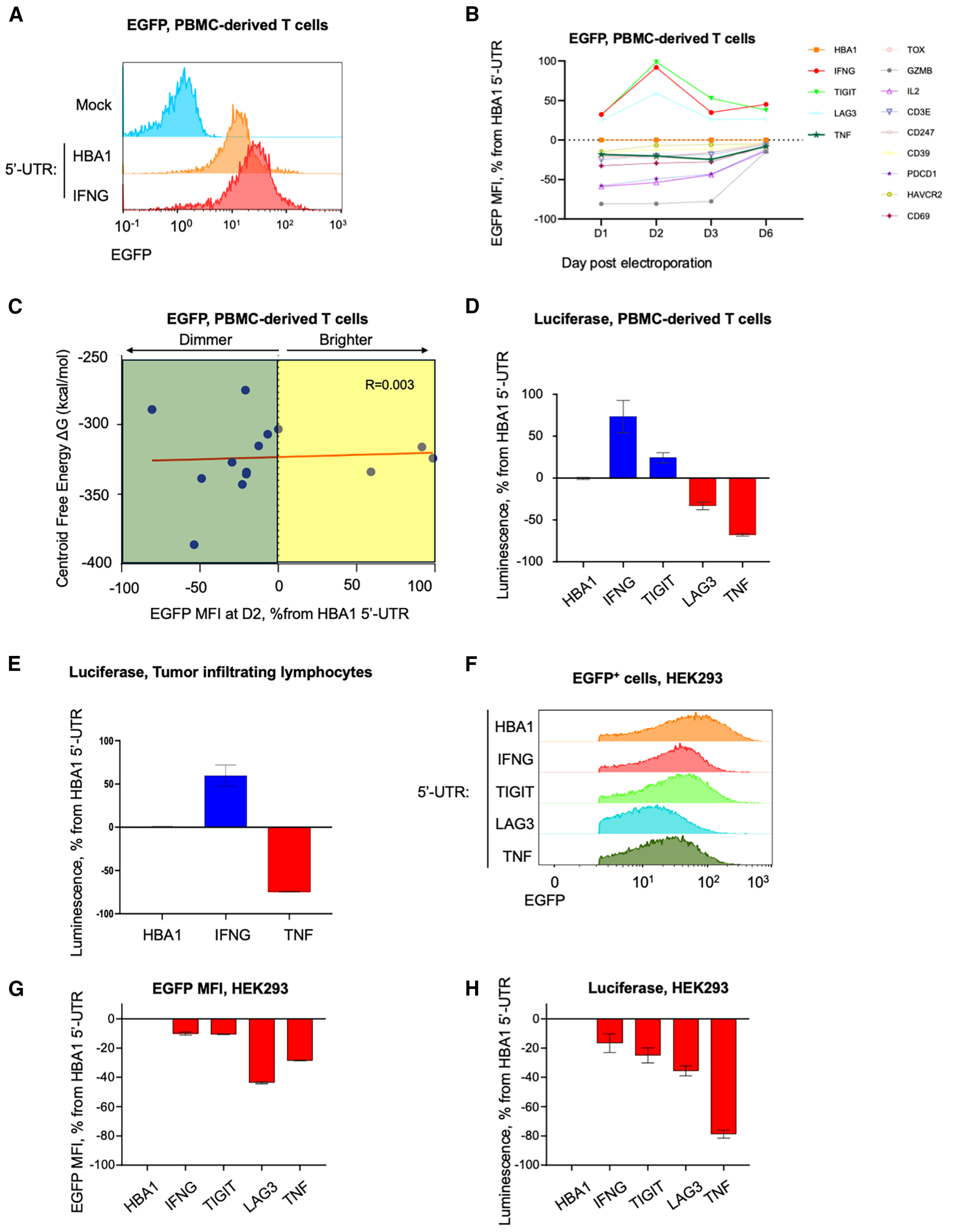

Figure 2. Modulation of gene reporter expression using T cell-specific UTRs

(A) Representative flow cytometry analysis of PBMC-derived T cells mock-electroporated or electroporated with EGFP-encoding constructs using either α-globin or *IFNG* 5′ UTR. Data are collected 24 h post-electroporation. Plots are representative of 3 independent donors. (B) Full analysis of PBMC-derived T cell EGFP MFI across days from electroporation. Data are relative to EGFP expression using HBA1 5′ UTR. (C) Plot of centroid minimal energy, corrected for pseudouridine usage as in Figure 1F, by normalized EGFP expression at day 2, compared with expression of *HBA1* 5′-UTR-*EGFP.* Correlation bar is in red. Note the low correlation. (D) Luciferase activity in PBMC-derived T cells electroporated with mRNA constructs harboring various 5′ UTRs, 24 h post-electroporation. Data are shown relative to the HBA1 control and represent mean ± SEM (n = 3). (E) Luciferase activity in melanoma-derived tumor-infiltrating lymphocytes electroporated with mRNA constructs harboring various 5′ UTRs.

(legend continued on next page)

compared to the *HBA1* 5′ UTR; instead, it was the *TIGIT* 5′ UTR that had the highest CD19-CAR expression among all engineered 5′ UTRs tested, although it was still slightly lower than that of the *HBA1* 5′ UTR. We decided to carry out functional experiments using only the *HBA1*, *TIGIT*, and *TNF* 5′ UTRs. Twenty-four hours post-mRNA electroporation, T cells were co-cultured with NALM6 $CD19^+$ target cells at different effector/target cell ratios and IFN-γ secretion was measured as output. Surprisingly, despite having lower CD19-CAR expression compared to *HBA1* 5′ UTR, the *TIGIT* 5′ UTR had slightly higher IFN-γ secretion. In contrast, *TNF* 5′ UTR had the lowest, as was evident in two different healthy PBMC donors (donors 29 and 40) (Figure 3D).

CAR signaling in the absence of antigen (tonic signaling) is a key obstacle in CAR biology, which drives CAR-T cells toward exhaustion.[18] To test whether the 5′ UTR impacts tonic signaling, we co-cultured different 5′-UTR-CAR-T cells with NALM6 $CD19^+$ vs. $CD19^-$ (NALM6-CD19ko)[19] cells at a high ratio of effector-to-target (E:T) ratio of 4:1 for 24 h. We confirmed the higher IFN-γ production from TIGIT 5′ UTR mRNA CAR-T in the presence of the target, and also saw that under these higher E:T ratios the TNF 5′ UTR mRNA CAR-T secreted IFN-γ similarly to HBA1 5′ UTR. When assessing for *IFNG* production in the absence of a target, representing tonic signaling, both *HBA1* 5′ UTR and *TIGIT* 5′ UTR constructs had similar high off-target output, whereas the *TNF* 5′ UTR construct led to minimal IFN-γ secretion even under these harsh terms (Figure 3E). This demonstrates the significance of the 5′ UTR also on tonic signaling in CAR-T models. To further confirm our findings, we used antigen-stimulated T cells previously activated in the presence of viral peptide pools, which have a higher activity/exhaustion profile compared to freshly isolated PBMCs. These viral-specific T cells (VSTs) were further electroporated using our system. A hallmark of tonic signaling due to excessive signaling of CARs in the absence of a target is increased exhaustion, manifested by elevated expression of checkpoint co-inhibitory molecules.[18] The VSTs have a high basal expression of PD-1, likely due to their excessive exposure to viral antigens, but do not express TIM-3. Within 24 h of electroporation of the HBA1-UTR-based CD19-CAR, TIM-3 expression was significantly increased. TIM-3 did not increase following the use of TIGIT and TNF-5′ UTR for CAR expression. Furthermore, the fraction of $PD\text{-}1^+TIM\text{-}3^+$ double-positive cells, known in the literature as deeply exhausted cells,[20] was lower in mRNA CAR expression following 5′ UTR from TIGIT of TNF compared to HBA1 (Figures 3F and 3G).

DISCUSSION

This study demonstrates that modifying the 5′ UTR of mRNA enables differential gene expression in human T cells. Replacing conventional HBA1 UTRs with sequences from highly expressed T cell genes allowed for fine-tuning of protein levels, thereby optimizing T cell engineering. A debate in the field of mRNA design revolves around the question of the contribution of UTRs and/or engineered ORFs by means of codon optimization, as was recently demonstrated for SARS-CoV-2 vaccines,[21] and some researchers even claim that ORF engineering outperforms UTR engineering. Our findings suggest that UTR engineering alone, while retaining the ORF, can modulate the expression of the mRNAs in a manner unpredicted by secondary RNA prediction; we therefore provide proof of concept for the modulation of 5′ UTR engineering on translation, compatible with other reports emphasizing the importance of 5′ UTR in translation efficiency.[22] Our data suggest that the UTRs of some genes, such as TNF, appear to act universally by reducing the expression of all genes of interest tested, regardless of the ORFs. In contrast, others, such as *IFNG* and *TIGIT* UTRs, modulate certain genes of interest in different ways; for instance, *IFNG* UTR elevated luciferase expression by 2-fold, but slightly reduced expression of CD19-CAR. In the context of CD19-CAR, the *TIGIT* 5′ UTR seems to both elevate reactivity toward target cells (demonstrated in Figure 3D) while maintaining relatively low tonic signaling and exhaustion (Figures 3F–3H), indicating that T cell-specific UTRs can modulate functional genes as well. Our observations suggest that different UTRs can interact with distinct ORFs to produce varying modulations of expression. Finally, the inability to predict 5′ UTR modulation of expression from RNA stability suggests that each 5′ UTR-ORF combination must be tested empirically in “wet” conditions.

With an increase in the use of mRNA-encoded T cell therapeutics in the clinic, UTR selection may improve the safety of CAR-T therapies, benefiting non-viral gene delivery and extending to other immune cell therapies. This may allow modulation of expression, control of on-target toxicity, and minimization of tonic signaling. These UTRs can also be used for other T cell-based applications, such as T cell reprogramming and TCR therapy.[23,24] Future studies should explore the mechanisms underlying UTR influence using ribosome profiling and RNA structure analysis and further improve the expression levels with modification of selected 3′ UTRs. *In vivo* validation is necessary to confirm the improved persistence and efficacy of CAR-T. Despite challenges such as transient mRNA expression and *in vivo* stability, advances in LNP delivery could enhance the therapeutic potential. The mRNA-based CAR system presented here is highly flexible and dynamic and can be used further as a screen platform for optimized CAR constructs.

In conclusion, our study establishes a novel approach for optimizing mRNA-based gene expression in T cells by leveraging endogenous

Data are shown relative to the HBA1 control and represent mean ± SEM (n = 3). (F) Representative flow cytometry analysis of HEK293 cells electroporated with mRNA constructs harboring various 5′ UTRs. (G) Full analysis of HEK293 cell *EGFP* MFI 24 h post-electroporation. Data are relative to EGFP expression using *HBA1* 5′ UTR. Data represent mean ± SEM (n = 2). (H) Full analysis of HEK293 cell luciferase 24 h post-electroporation. Data are relative to luciferase activity using HBA1 5′ UTR. Data are normalized to HBA1 and presented as mean ± SEM (n = 2).

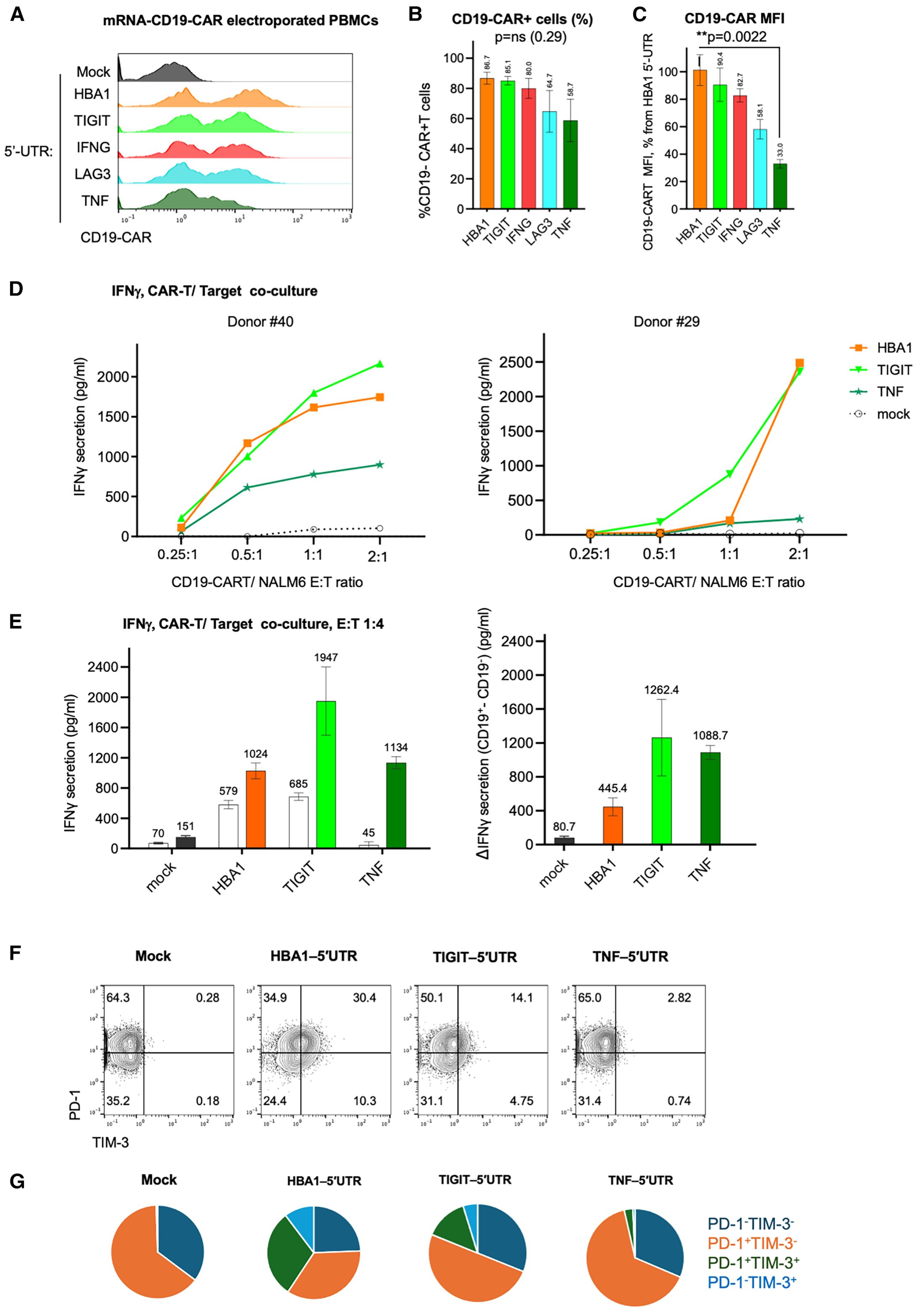

(legend on next page)

UTR sequences. By demonstrating that UTR selection can modulate protein expression and functional signaling, we provide a valuable tool for rationalizing next-generation engineered T cell therapies. Future work should focus on expanding this platform to additional gene targets and validating its therapeutic potential in clinical models of cancer immunotherapy.

MATERIALS AND METHODS

RNA secondary structure predictions

RNA structures were predicted using the RNAfold webserver (http://rna.tbi.univie.ac.at/cgi-bin/RNAWebSuite/RNAfold.cgi). Minimal free energy for the centroid structure, as well as mountain plots, was downloaded following the analysis.

Cell preparation

Peripheral blood-derived T cells were isolated from PBMCs using the Pan T cell Isolation Kit (Miltenyi Biotec, #130-096-535) with LS columns and a MACS Separator, following the manufacturer's manual magnetic labeling protocol. Reagents were kept at 4°C, and volumes were scaled for 200 × 10^6 PBMCs. Unlabeled $CD3^+$ T cells were collected, washed with PBS, and resuspended in serum-free RPMI.

Cells were cultured overnight in T2 medium (RPMI supplemented with 10% fetal bovine serum (FBS), 25 mM HEPES buffer, 100 U/mL penicillin/streptomycin, 2 mM L-glutamine, and 1 mM Na-pyruvate) with IL-2 (100 IU/mL) at 150 × 10^6 cells per upright flask (4 flasks, 600 × 10^6 total). Yield (∼30% of input; 92% viability) was confirmed by automated cell counter and flow cytometry using anti-CD3, CD4, and CD8 staining.

Tumor infiltrating lympocytes (TILs) were obtained from an immortalized source and processed similarly as described previously.[25]

Electroporation procedure

In vitro-transcribed (IVT) mRNAs encoding CD19-CAR, EGFP, luciferase, or control constructs were synthesized using the HiScribe T7 ARCA mRNA Kit (NEB), incorporating an ARCA cap, 5′/3′ UTRs, and a poly(A) tail. Electroporation was performed under sterile, RNase-free conditions using the Gene Pulser Xcell system (Bio-Rad) in square-wave mode (400 V, 1.5 ms, unless otherwise noted). Optimization tests showed that 400 V/1.5 ms yielded robust GFP expression; lower voltage/duration (250 V/10 ms) resulted in reduced expression. No signal was detected in mock controls. T cells and HEK293 cells (>90% viability) were washed with cold Opti-MEM and resuspended at 2 × 10^6 cells/100 μL. TILs (64% viability) were prepared at 4.5 × 10^6 cells/300 μL. Electroporation was performed in 2-mm cuvettes using 1–5 μg IVT mRNA per 100 μL. For 5′ UTR screening, constructs were tested in triplicate at 1, 2.5, and 5 μg; HBA1 5′ UTR (5 μg) served as control. Mock-electroporated cells (no RNA) were included. Following electroporation, cells were transferred to 24-well plates with 2 mL T2 medium containing IL-2 (100 IU/mL) and incubated at 37°C. GFP or CD19-CAR expression and retention of surface markers (e.g., TIM-3) were assessed by flow cytometry after 24 h. For co-electroporation, total RNA amounts were normalized using irrelevant IVT mRNA.

Plasmid design and *in vitro* transcription of mRNA

mRNA constructs were designed as synthetic modRNA (modified mRNA) containing a 5′ ARCA cap, a variable 5′ UTR, a Kozak consensus sequence, an ORF encoding a gene of interest (GFP, luciferase, or CD19-CAR), a 3′ UTR derived from the human hemoglobin alpha-1 gene (HBA1), and a poly(A) tail consisting of two 60-adenine stretches separated by a single guanine (60 A-G-60 A). To identify UTR sequences enhancing mRNA translation in T cells, a synthetic 5′ UTR library was constructed. Candidate sequences were selected based on expression in T cell subsets and known translational regulators. Genes included HBA1, CD3ζ, checkpoint molecules, and cytokine receptors. Each UTR was fused to a GFP or luciferase ORF with a fixed 3′ UTR and poly(A) tail. Constructs were cloned into plasmids with T7 promoters and PmeI sites for IVT. Following transcription, mRNAs were electroporated into human T cells. The expression was assessed by flow cytometry (GFP), luminescence (luciferase), and cytokine assays (MSD) to quantify protein expression and function.

Plasmid linearization

Plasmids were linearized using the PmeI restriction enzyme. Each reaction included 50 μg plasmid DNA, 50 μL of 10× Smart Cut Buffer, 1–1.5 μL PmeI, and RNase-free water to 500 μL. Samples were incubated at 37°C overnight. DNA was precipitated by adding 1/10 volume of 5 M ammonium acetate and 2 volumes of 100% ethanol, incubated at −20°C, centrifuged, washed with 70% ethanol, and resuspended

Figure 3. T cell-specific UTRs for optimized CAR expression, reactivity, and tonic signaling

(A). Representative flow cytometry analysis of PBMC-derived T cells electroporated with CD19-CAR-encoding constructs using various 5′ UTRs. (B) Quantification of percentages of CD19-CAR^+ cells in (A). Data are normalized to the HBA1 UTR condition and presented as mean ± SEM from four independent experiments ($n = 4$). Kruskal-Wallis test revealed no statistically significant difference ($p = 0.29$). (C) MFI of CD19-CAR^+ cells in (A) normalized to the HBA1 UTR. Data represent four independent experiments ($n = 4$). Kruskal-Wallis test indicated a significant difference among groups ($p < 0.005$); Dunn's post hoc test revealed significant reduction in the TNF-UTR group compared to HBA1 ($p = 0.0022$). (D) Secreted IFN-γ levels in co-culture supernatants of PBMC-derived T cells electroporated with CD19-CAR mRNA using different 5′ UTRs and $CD19^+$ NALM6 target cells. Data from two healthy donors (D29 and D40) are shown at various effector-to-target (E:T) ratios. (E) Left: interferon gamma ELISA in media taken from co-cultures of PBMC-derived T cells electroporated with CD19-CAR mRNA using different UTRs, either together with $CD19^+$ (filled) or $CD19^-$ (NALM6 KO, empty) at an E:T ratio of 4:1. Right: delta of interferon gamma secretion of Figure 2E between the co-culture of electroporated T cells with $CD19^+$ vs. $CD19^-$ NALM6 cells. Bars represent mean ± SEM, $n = 5$ ($CD19^+$ co-cultures) and $n = 2$ ($CD19^-$ co-cultures) per construct. (F) Flow cytometry analysis of virus-specific T cells (VSTs) electroporated with mRNA constructs encoding for CD19-CAR using various UTRs. Note that cells are grown without target cells to demonstrate tonic signaling. (G) Pie charts of PD-1/TIM-3 population distribution of (F). Data are representative of two independent experiments.

in 50 μL pre-warmed RNase-free water. Linearization was confirmed on a 1% agarose gel, and purity was verified by NanoDrop.

In vitro transcription and nucleotide modification

IVT was performed using the HiScribe T7 ARCA mRNA Kit (NEB #E2065S) enriched with pseudouridine-UTP (Ψ-UTP). Each 20 μL reaction contained 10 μL ARCA/NTP mix, 2.5 μL Ψ-UTP, ~1 μg linearized DNA, 2 μL T7 RNA polymerase mix, and water to volume. Reactions were incubated at 37°C for 4 h, followed by DNase I treatment. Pseudouridine is ~70% enriched under these conditions.

mRNA purification and storage

Each IVT reaction was diluted with 30 μL water and 25 μL LiCl solution, incubated at -20°C, and centrifuged. The RNA pellet was washed with 70% ethanol, air dried, and resuspended in 50 μL 0.1 mM EDTA. Samples were heated to 65°C to fully dissolve and stored at -20°C.

Flow cytometry and cytokine assays

Flow cytometry was used to assess the expression of reporter genes (GFP), surface molecules (CD19-CAR), and exhaustion/activation markers (e.g., TIM-3, 4-1BB, OX40, and PD-1). Staining was performed on equal numbers of viable cells (200,000 per sample), as determined by cell counting using an automated cell counter. Cells were harvested 24–48 h post-electroporation, washed in FACS buffer (PBS with 2% FBS), and stained with the following fluorochrome-conjugated antibodies: CD19 CAR Detection Reagent, Biotin (Miltenyi Biotec, #130-129-550), followed by secondary staining with Anti-Biotin-APC (Miltenyi Biotec, REAfinity #130-113-854); TIM-3 APC-Cy7 (BioLegend, #345025); 4-1BB PE-Cy7 (BioLegend, #309820); Viability Dye eFluor 506/AmCyan (Thermo Fisher Scientific, #65-0866-14). Cells were first stained with surface antibodies and then viability staining was performed. An unstained control stained only with viability dye was included. To generate a dead-cell control, cells were heated at 60°C for 10 min and stained with the full panel. Samples were acquired on a Miltenyi flow cytometer and analyzed with FlowJo software. Expression levels were quantified as percent positive and mean fluorescence intensity (MFI). To assess cytokine secretion, electroporated T cells were co-cultured with $CD19^+$ or $CD19^-$ NALM6 target cells at defined E:T ratios (0.25, 0.5:1, 1:1, 2:1, 4:1) for 24 or 48 h, depending on the assay. Supernatants were collected and frozen at −80°C until analysis. IFN-γ was quantified using ELISA kits (BioLegend).

Luciferase assay

Luciferase activity was assessed 24 h post-electroporation. Cells were lysed in Passive Lysis Buffer (Promega) and analyzed using the Luciferase Assay System (Promega). Luminescence was measured using a plate reader (e.g., BioTek Synergy), and results were normalized to cell number or protein concentration.

Cell culture and target cell co-culture

Primary human T cells, VSTs, and TILs were cultured in RPMI 1640 medium supplemented with 10% heat-inactivated FBS, 2 mM L-glutamine, 1% penicillin-streptomycin, and 100 IU/mL recombinant human IL-2. Cells were maintained at 37°C and 5% CO_2. VSTs were activated with antigen-presenting cells loaded with a pool of viral peptides for 10 days, harvested, and frozen until experiments. To assess cytotoxicity, CD19-CAR-transfected T cells (5′ UTR variants or mock) were co-cultured with $CD19^+$ or CD19-KO NALM6 cells in U-bottom 96-well plates. Transfected T cells were counted 20 h post-electroporation and plated in 100 μL T2 medium with 300 IU/mL IL-2 at 80,000, 40,000, 20,000, 10,000, and 5,000 cells/well, plus a no-effector control. NALM6 cells (30,000/well) were added to achieve E:T ratios from 5:1 to 1:5. Each condition was tested in quadruplicate; CD19-KO cells were included as non-target controls. After 24 h, 100 μL supernatants were collected from triplicate wells for IFN-γ quantification by ELISA. Cells were stained for CD3 and CD10 and analyzed by flow cytometry to assess T cell presence and target cell killing.

T cell functional assays

Functional evaluation of transfected T cells, either isolated from peripheral blood mononuclear cells (PBMCs) or VSTs, was conducted through co-culture with wild-type $CD19^+$ NALM6 cells or CD19-knockout (NALM6-CD19-ko) NALM6 cells. Effector cells (PBMC-derived T cells or VSTs) were plated in technical triplicates with target cells at defined E:T ratios of 1:1, 2:1, or 4:1 in round-bottom 96-well plates. After 24 h of co-culture, supernatants were collected for cytokine analysis using ELISA or MSD assays, and cells were harvested for flow cytometry to evaluate CAR expression and activation/exhaustion marker expression.

Statistical analysis

Statistical analyses were performed in GraphPad Prism (v.9+). Kruskal-Wallis tests with Dunn's post hoc correction were used for multiple comparisons; Wilcoxon matched-pairs signed rank test was used for paired data. Data are shown as mean ± SEM. Significance was accepted at $p < 0.05$. Each experiment was independently repeated and included at least two biological replicates.

DATA AND CODE AVAILABILITY

The data generated in this study are available from the corresponding authors upon reasonable request. Source data underlying all figures, including flow cytometry (.fcs) files, luciferase activity measurements, ELISA cytokine quantifications, and RNA secondary structure prediction outputs (RNAfold), are available upon request.

Plasmid maps and full sequences of the engineered 5′ UTRs used in the mRNA constructs have been deposited in a publicly accessible repository and are available upon reasonable request, subject to institutional material transfer agreements. Any custom analysis scripts used for RNA free-energy correction calculations are available from the corresponding authors upon request.

ACKNOWLEDGMENTS

G.C. is supported by a research grant from Pfizer, research grant from the Israeli Ministry of Science and Technology, the Kamin program from the Israel Innovation

authority, research grant from Sheba Medical Center, and the Weizmann Institute, research grant from the Israel Cancer Association. Y.W. is supported by a Melanoma Research Alliance grant (no. 937368), the Rosetrees Trust (no. MYIA\100002), a research grant from Pfizer, Dotan Center for Hematologic Research, Israel Cancer Research Foundation, and the Lemelbaum family. E.J. is supported by the Dotan Center for Hematologic Research and the Israel Cancer Association grant.

AUTHOR CONTRIBUTIONS

G.G., N.T., A.M., S.K., S.A., and O.H. designed and performed experiments. A.S., H.A.-H., O.I., R.S.-F., and E.J. provided useful insights and consultation. G.C., E.J., and Y.W. designed mRNA constructs and provided funding. G.G., G.C., and Y.W. conceived the research, analyzed data, and wrote the paper.

DECLARATION OF INTERESTS

G.C. and Y.W. receive a research grant from Pfizer, which is unrelated to this work. G.C., G.G., and Y.W. are in the process of writing a patent on T cell-specific mRNAs, based on this work.

SUPPLEMENTAL INFORMATION

Supplemental information can be found online at https://doi.org/10.1016/j.omtn.2026.102913.

REFERENCES

1. Warren, L., and Lin, C. (2019). mRNA-Based Genetic Reprogramming. Mol. Ther. *27*, 729–734. https://doi.org/10.1016/j.ymthe.2018.12.009.

2. Tavernier, G., Andries, O., Demeester, J., Sanders, N.N., De Smedt, S.C., and Rejman, J. (2011). mRNA as gene therapeutic: how to control protein expression. J. Contr. Release *150*, 238–247. https://doi.org/10.1016/j.jconrel.2010.10.020.

3. Plews, J.R., Li, J., Jones, M., Moore, H.D., Mason, C., Andrews, P.W., and Na, J. (2010). Activation of pluripotency genes in human fibroblast cells by a novel mRNA based approach. PLoS One *5*, e14397. https://doi.org/10.1371/journal.pone.0014397.

4. Sultana, N., Hadas, Y., Sharkar, M.T.K., Kaur, K., Magadum, A., Kurian, A.A., Hossain, N., Alburquerque, B., Ahmed, S., Chepurko, E., and Zangi, L. (2020). Optimization of 5' Untranslated Region of Modified mRNA for Use in Cardiac or Hepatic Ischemic Injury. Mol. Ther. Methods Clin. Dev. *17*, 622–633. https://doi.org/10.1016/j.omtm.2020.03.019.

5. Orlandini von Niessen, A.G., Poleganov, M.A., Rechner, C., Plaschke, A., Kranz, L.M., Fesser, S., Diken, M., Löwer, M., Vallazza, B., Beissert, T., et al. (2019). Improving mRNA-Based Therapeutic Gene Delivery by Expression-Augmenting 3' UTRs Identified by Cellular Library Screening. Mol. Ther. *27*, 824–836. https://doi.org/10.1016/j.ymthe.2018.12.011.

6. Castillo-Hair, S., Fedak, S., Wang, B., Linder, J., Havens, K., Certo, M., and Seelig, G. (2024). Optimizing 5'UTRs for mRNA-delivered gene editing using deep learning. Nat. Commun. *15*, 5284. https://doi.org/10.1038/s41467-024-49508-2.

7. Billingsley, M.M., Gong, N., Mukalel, A.J., Thatte, A.S., El-Mayta, R., Patel, S.K., Metzloff, A.E., Swingle, K.L., Han, X., Xue, L., et al. (2024). In Vivo mRNA CAR T Cell Engineering via Targeted Ionizable Lipid Nanoparticles with Extrahepatic Tropism. Small *20*, e2304378. https://doi.org/10.1002/smll.202304378.

8. Li, Y., Tian, Y., Li, C., Fang, W., Li, X., Jing, Z., Yang, Z., Zhang, X., Huang, Y., Gong, J., et al. (2024). In situ engineering of mRNA-CAR T cells using spleen-targeted ionizable lipid nanoparticles to eliminate cancer cells. Nano Today *59*, 102518. https://doi.org/10.1016/j.nantod.2024.102518.

9. Liu, H., Yin, J., Xiao, M., Gao, C., Mason, A.S., Zhao, Z., Liu, Y., Li, J., and Fu, D. (2012). Characterization and evolution of 5' and 3' untranslated regions in eukaryotes. Gene *507*, 106–111. https://doi.org/10.1016/j.gene.2012.07.034.

10. Nanduri, S., Carpick, B.W., Yang, Y., Williams, B.R., and Qin, J. (1998). Structure of the double-stranded RNA-binding domain of the protein kinase PKR reveals the molecular basis of its dsRNA-mediated activation. EMBO J. *17*, 5458–5465. https://doi.org/10.1093/emboj/17.18.5458.

11. Gruber, A.R., Lorenz, R., Bernhart, S.H., Neuböck, R., and Hofacker, I.L. (2008). The Vienna RNA websuite. Nucleic Acids Res. *36*, W70–W74. https://doi.org/10.1093/nar/gkn188.

12. Anderson, B.R., Muramatsu, H., Nallagatla, S.R., Bevilacqua, P.C., Sansing, L.H., Weissman, D., and Karikó, K. (2010). Incorporation of pseudouridine into mRNA enhances translation by diminishing PKR activation. Nucleic Acids Res. *38*, 5884–5892. https://doi.org/10.1093/nar/gkq347.

13. Boo, S.H., and Kim, Y.K. (2020). The emerging role of RNA modifications in the regulation of mRNA stability. Exp. Mol. Med. *52*, 400–408. https://doi.org/10.1038/s12276-020-0407-z.

14. Karikó, K., Buckstein, M., Ni, H., and Weissman, D. (2005). Suppression of RNA recognition by Toll-like receptors: the impact of nucleoside modification and the evolutionary origin of RNA. Immunity *23*, 165–175. https://doi.org/10.1016/j.immuni.2005.06.008.

15. Nallagatla, S.R., Hwang, J., Toroney, R., Zheng, X., Cameron, C.E., and Bevilacqua, P.C. (2007). 5'-triphosphate-dependent activation of PKR by RNAs with short stem-loops. Science *318*, 1455–1458. https://doi.org/10.1126/science.1147347.

16. Bevilacqua, P.C., and Cech, T.R. (1996). Minor-groove recognition of double-stranded RNA by the double-stranded RNA-binding domain from the RNA-activated protein kinase PKR. Biochemistry *35*, 9983–9994. https://doi.org/10.1021/bi9607259.

17. Itzhaki, O., Jacoby, E., Nissani, A., Levi, M., Nagler, A., Kubi, A., Brezinger, K., Brayer, H., Zeltzer, L.A., Rozenbaum, M., et al. (2020). Head-to-head comparison of in-house produced CD19 CAR-T cell in ALL and NHL patients. J. Immunother. Cancer *8*, e000148. https://doi.org/10.1136/jitc-2019-000148.

18. Long, A.H., Haso, W.M., Shern, J.F., Wanhainen, K.M., Murgai, M., Ingaramo, M., Smith, J.P., Walker, A.J., Kohler, M.E., Venkateshwara, V.R., et al. (2015). 4-1BB costimulation ameliorates T cell exhaustion induced by tonic signaling of chimeric antigen receptors. Nat. Med. *21*, 581–590. https://doi.org/10.1038/nm.3838.

19. Rozenbaum, M., Meir, A., Aharony, Y., Itzhaki, O., Schachter, J., Bank, I., Jacoby, E., and Besser, M.J. (2020). Gamma-Delta CAR-T Cells Show CAR-Directed and Independent Activity Against Leukemia. Front. Immunol. *11*, 1347. https://doi.org/10.3389/fimmu.2020.01347.

20. Sakuishi, K., Apetoh, L., Sullivan, J.M., Blazar, B.R., Kuchroo, V.K., and Anderson, A.C. (2010). Targeting Tim-3 and PD-1 pathways to reverse T cell exhaustion and restore anti-tumor immunity. J. Exp. Med. *207*, 2187–2194. https://doi.org/10.1084/jem.20100643.

21. Zhang, H., Zhang, L., Lin, A., Xu, C., Li, Z., Liu, K., Liu, B., Ma, X., Zhao, F., Jiang, H., et al. (2023). Algorithm for optimized mRNA design improves stability and immunogenicity. Nature *621*, 396–403. https://doi.org/10.1038/s41586-023-06127-z.

22. Chu, Y., Yu, D., Li, Y., Huang, K., Shen, Y., Cong, L., Zhang, J., and Wang, M. (2024). A 5′ UTR language model for decoding untranslated regions of mRNA and function predictions. Nat. Mach. Intell. *6*, 449–460. https://doi.org/10.1038/s42256-024-00823-9.

23. Cafri, G., Gartner, J.J., Zaks, T., Hopson, K., Levin, N., Paria, B.C., Parkhurst, M.R., Yossef, R., Lowery, F.J., Jafferji, M.S., et al. (2020). mRNA vaccine-induced neoantigen-specific T cell immunity in patients with gastrointestinal cancer. J. Clin. Invest. *130*, 5976–5988. https://doi.org/10.1172/JCI134915.

24. Peri, A., Salomon, N., Wolf, Y., Kreiter, S., Diken, M., and Samuels, Y. (2023). The landscape of T cell antigens for cancer immunotherapy. Nat. Cancer *4*, 937–954. https://doi.org/10.1038/s43018-023-00588-x.

25. Itzhaki, O., Hovav, E., Ziporen, Y., Levy, D., Kubi, A., Zikich, D., Hershkovitz, L., Treves, A.J., Shalmon, B., Zippel, D., et al. (2011). Establishment and large-scale expansion of minimally cultured "young" tumor infiltrating lymphocytes for adoptive transfer therapy. J. Immunother. *34*, 212–220. https://doi.org/10.1097/CJI.0b013e318209c94c.

OMTN, Volume *37*

Supplemental information

Engineered mRNA backbones

for gene expression in human T cells

Gilad Gibor, Neve Tzvi, Amilia Meir, Hiba Abu-Hariri, Anat Shemer, Shai Kilim, Sophie Abelian, Ortal Harush, Orit Itzhaki, Ronnie Shapira-Frommer, Elad Jacoby, Gal Cafri, and Yochai Wolf

Supplemental Information

Table S1. All constructs used in the research

**5'-UTR**	***Accession number***	**GOI**
Globin (HBA1)	NM_00058	EGFP
IFN-gamma	NM_000619	EGFP
TIGIT	NM_173799	EGFP
LAG3	NM_002286	EGFP
TNF	NM_000594	EGFP
TOX	NM_014729	EGFP
GNZB	NM_004131	EGFP
IL-2	NM_000586	EGFP
CD3-epsilon	NM_000733	EGFP
CD3-zeta (CD247)	NM_000734	EGFP
CD39	NM_001776	EGFP
PD1	NM_005018	EGFP
TIM-3	NM_032782	EGFP
CD69	NM_001781	EGFP
TIGIT		Luc
Globin		Luc
IFN-gamma		Luc
TNF		Luc
LAG3		Luc
Globin		CD19-CAR
IFN-gamma		CD19-CAR
TNF		CD19-CAR
LAG3		CD19-CAR
TIGIT		CD19-CAR

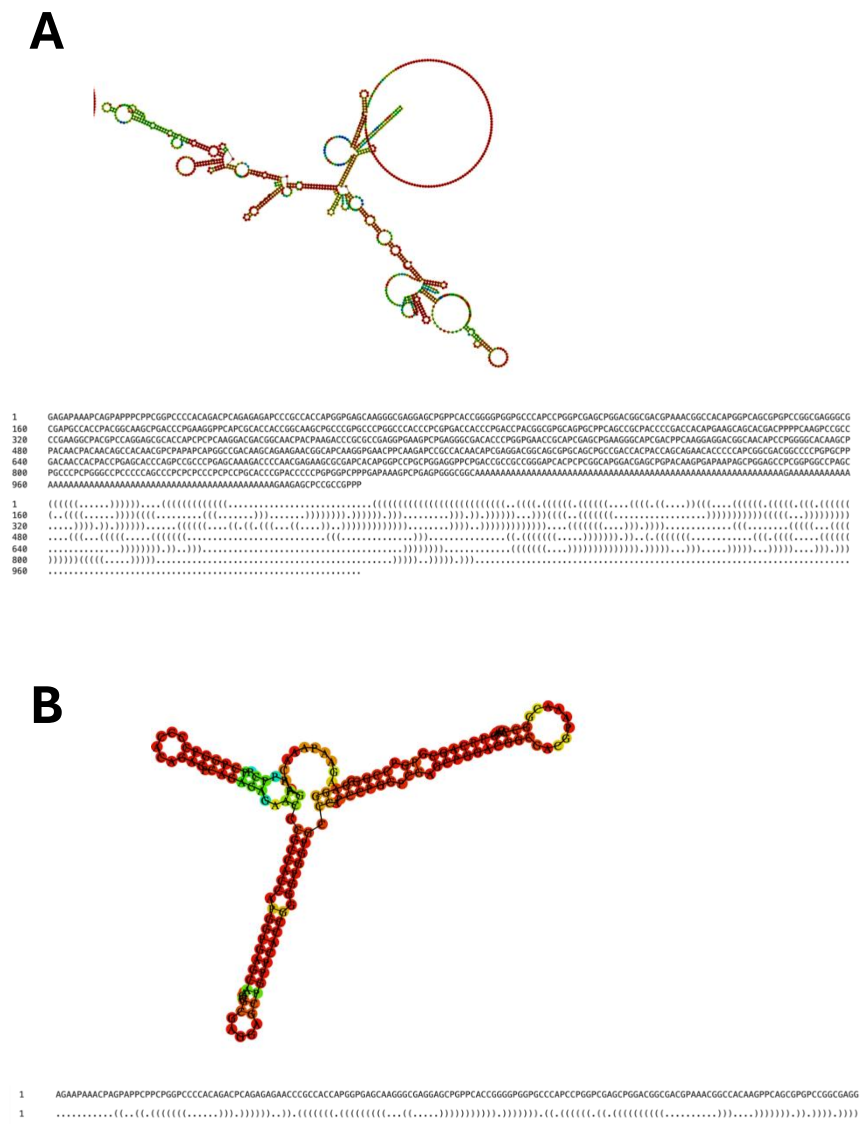

Figure S1 1. RNAFold analysis for 5’-HBA1-EGFP, both for its full structure (A) and its first 100 coding sequnce base pairs (B)

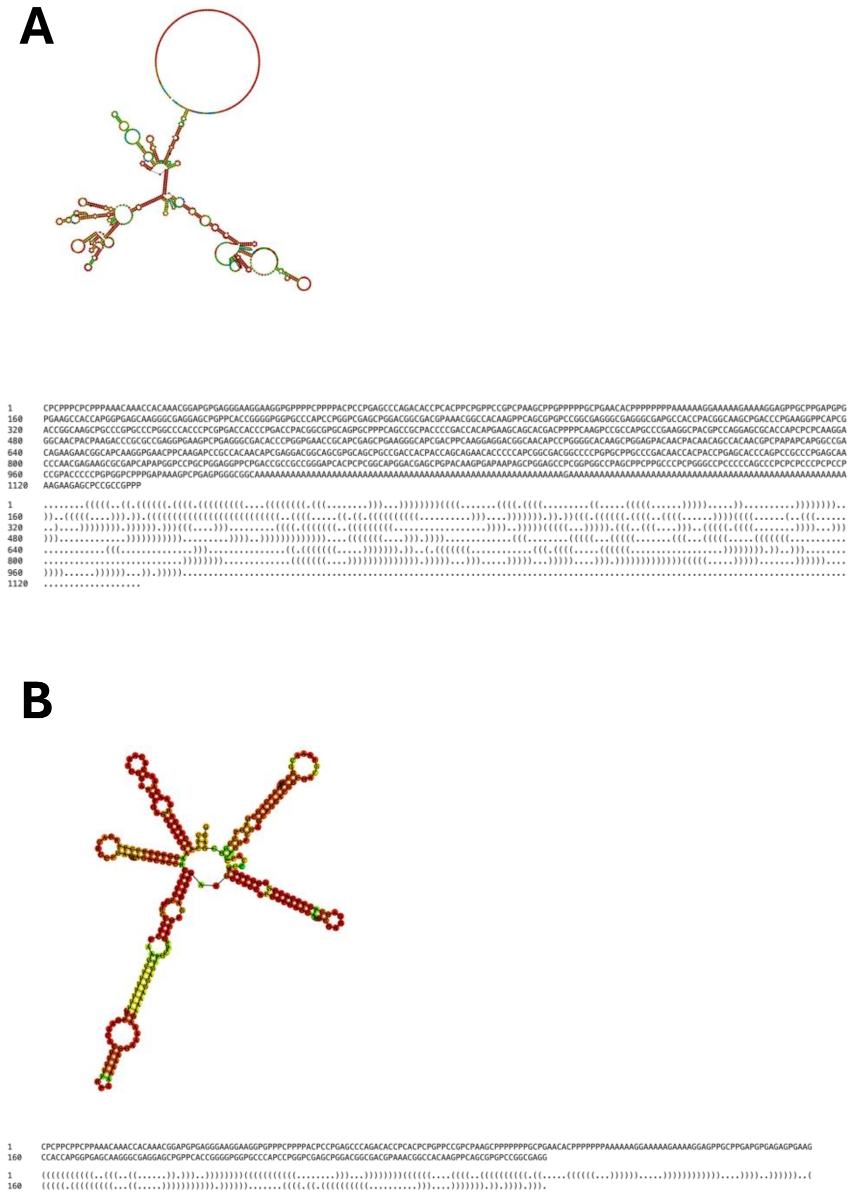

Figure S2. RNAFold analysis for 5’-TOX-EGFP, both for its full structure (A) and its first 100 coding sequnce base pairs (B)

A

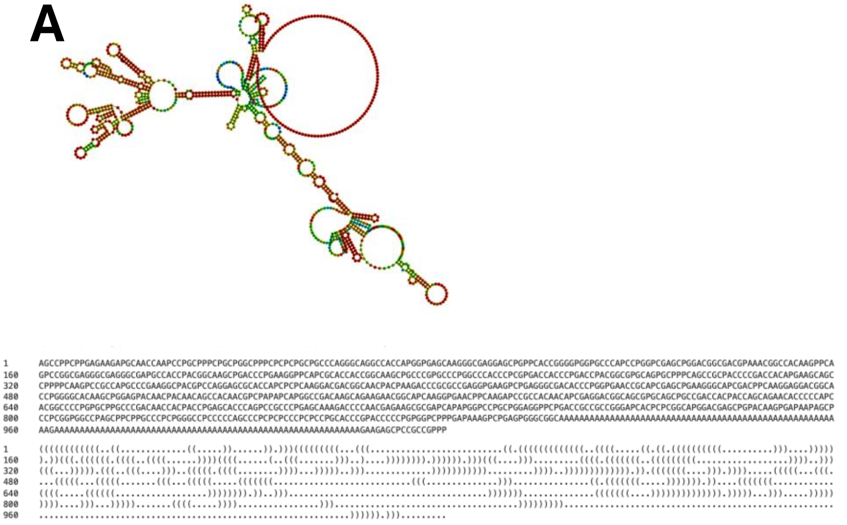

B

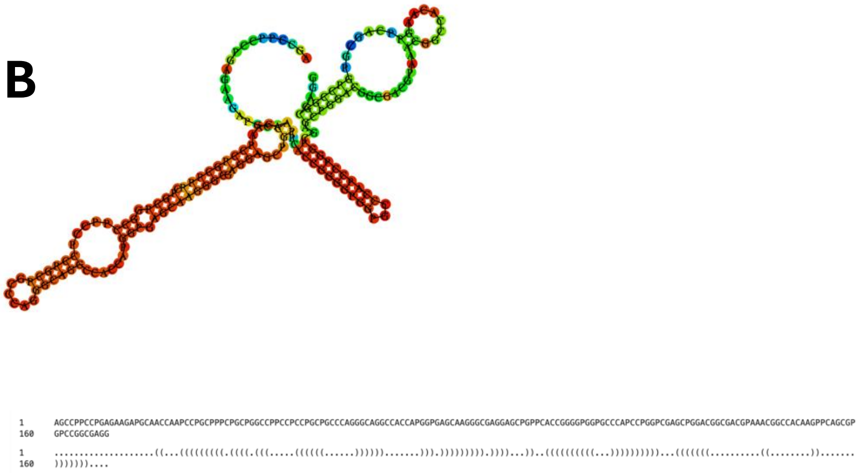

Figure S3. RNAFold analysis for 5'-GZMB-EGFP, both for its full structure (A) and its first 100 coding sequnce base pairs (B)

A

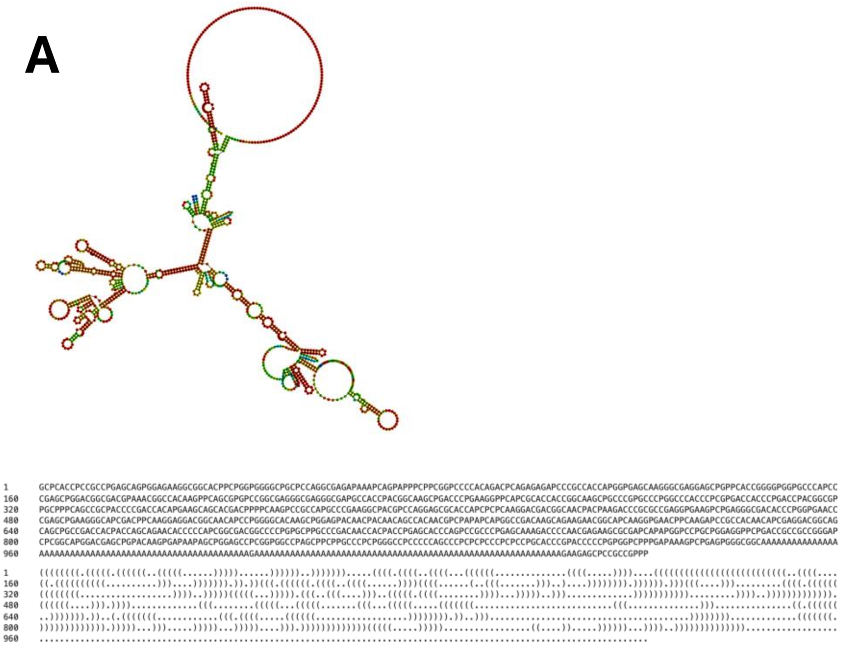

B

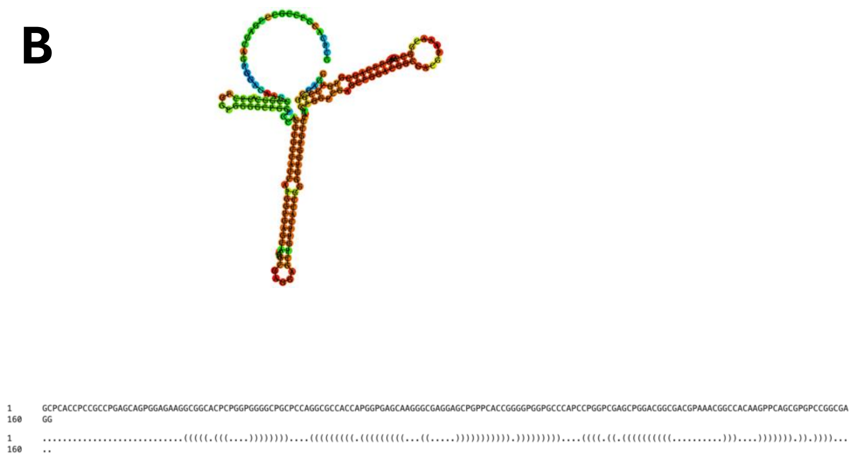

Figure S4. RNAFold analysis for 5'-PD1-EGFP, both for its full structure (A) and its first 100 coding sequnce base pairs (B)

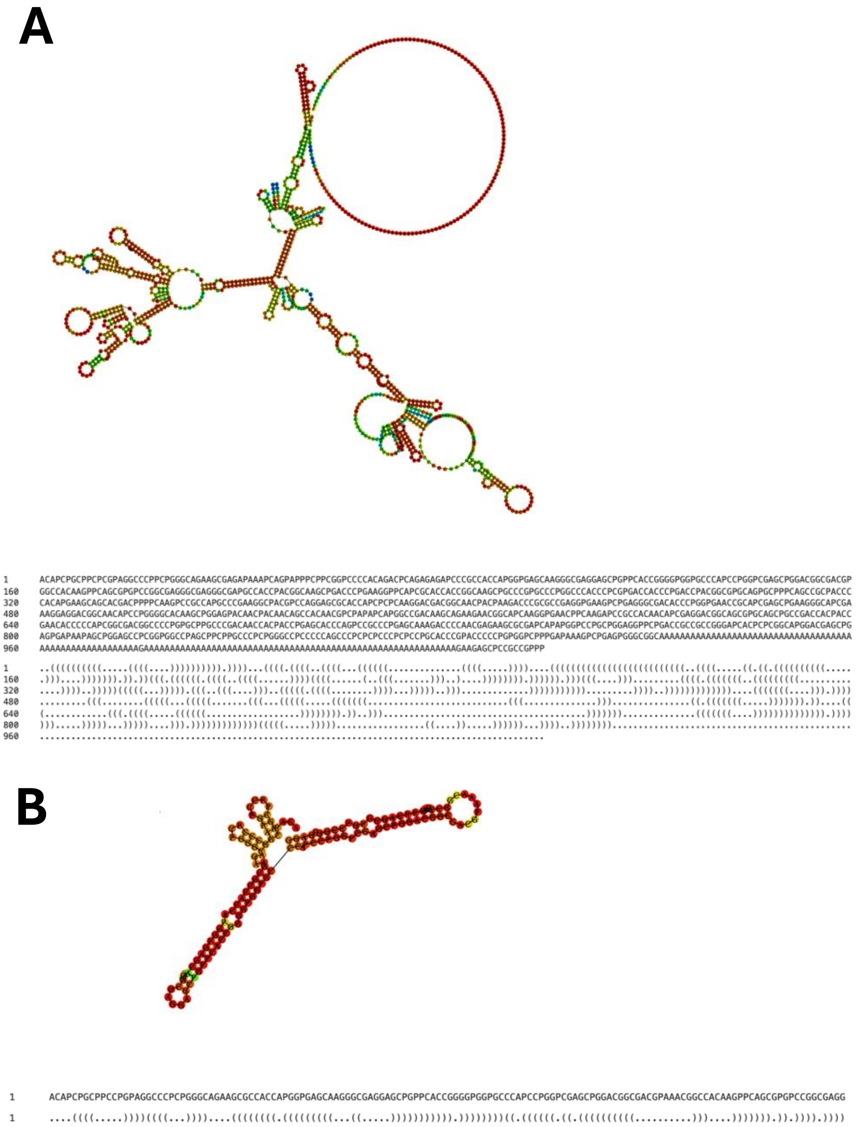

Figure S5. RNAFold analysis for 5'-TIGIT-EGFP, both for its full structure (A) and its first 100 coding sequnce base pairs (B)

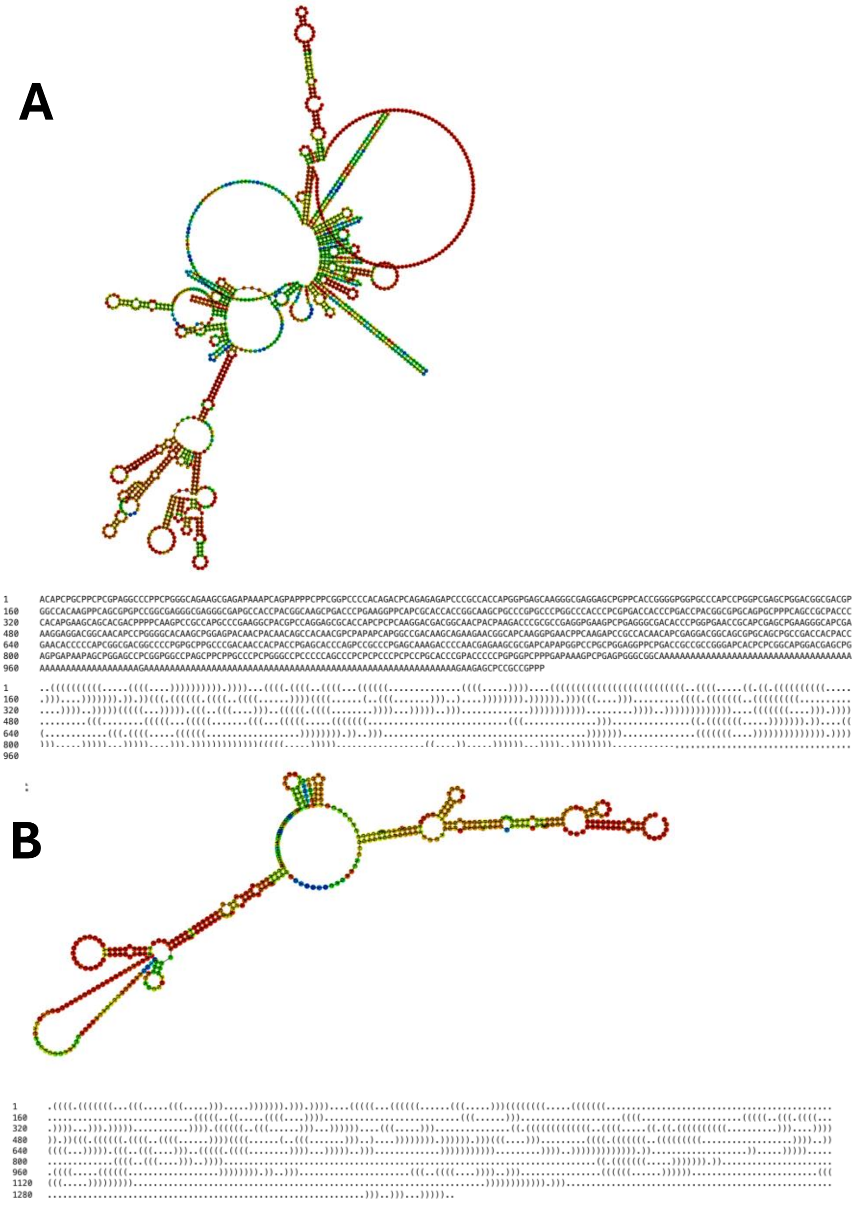

Figure S6. RNAFold analysis for 5'-LAG3-EGFP, both for its full structure (A) and its first 100 coding sequnce base pairs (B)

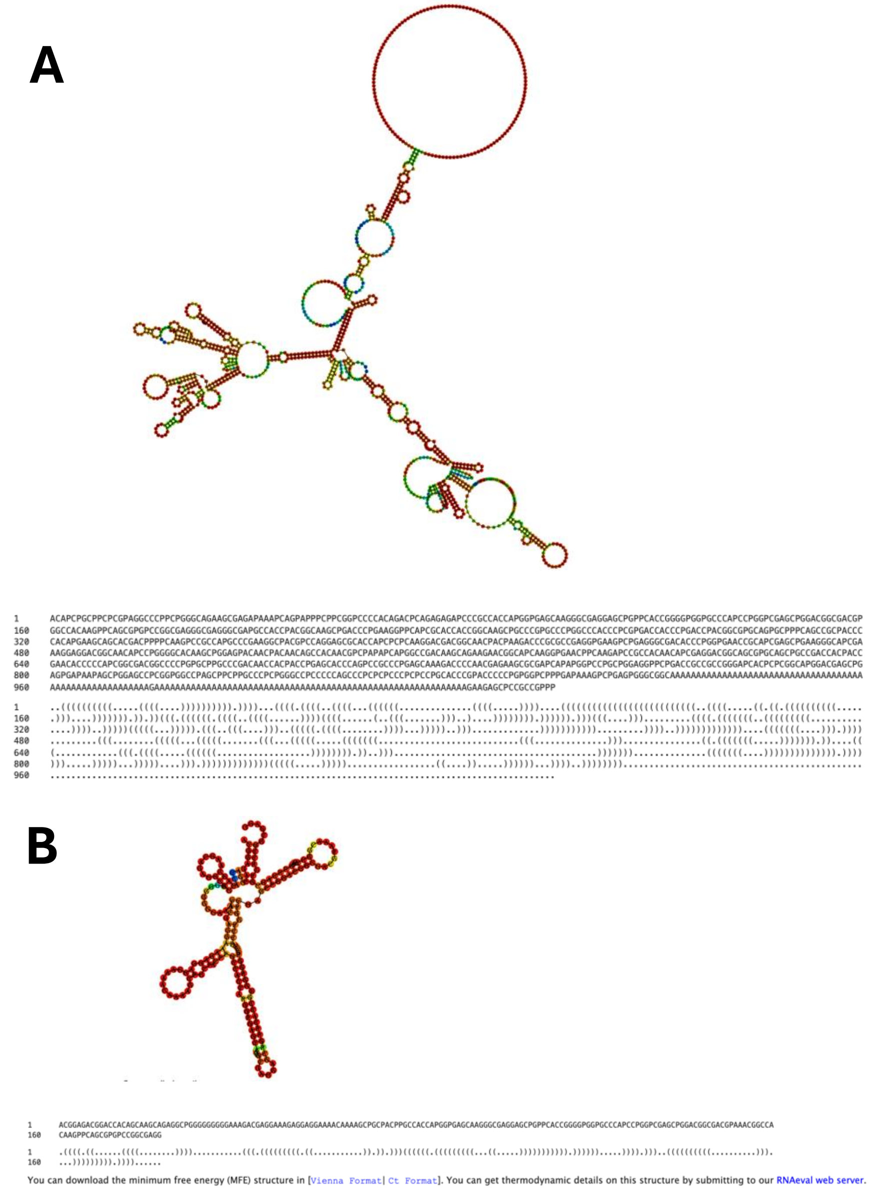

Figure S7. RNAFold analysis for 5’-CD39-EGFP, both for its full structure (A) and its first 100 coding sequnce base pairs (B)

A

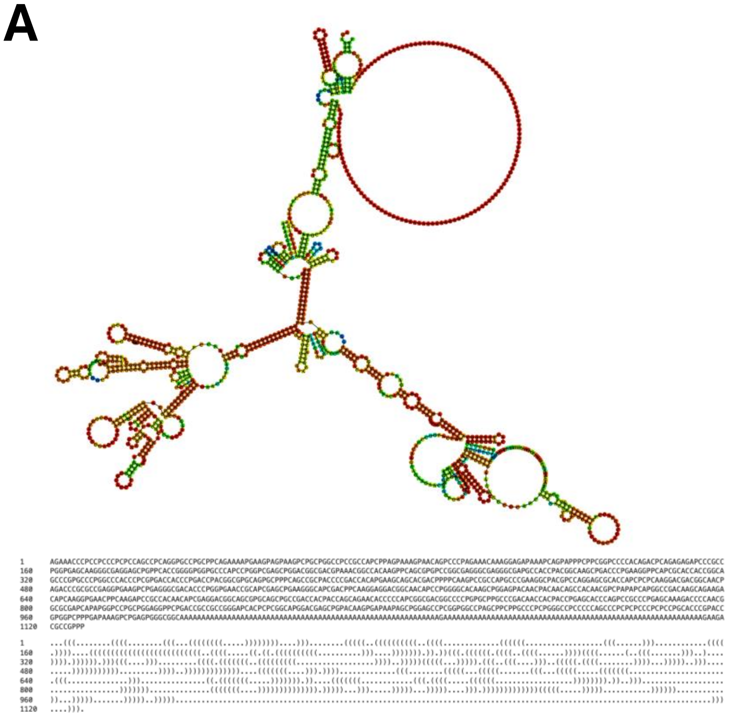

B

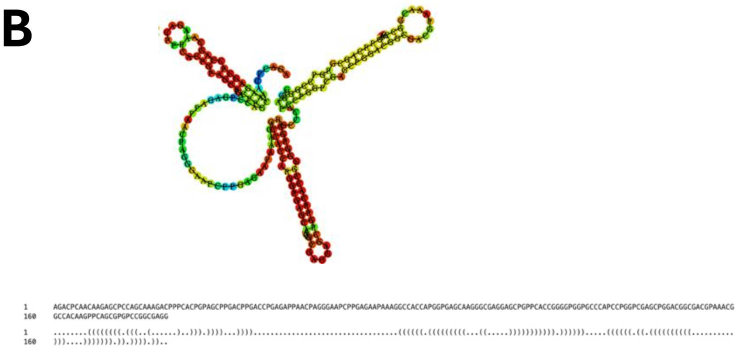

Figure S8. . RNAFold analysis for 5'-CD69-EGFP, both for its full structure (A) and its first 100 coding sequnce base pairs (B)

A

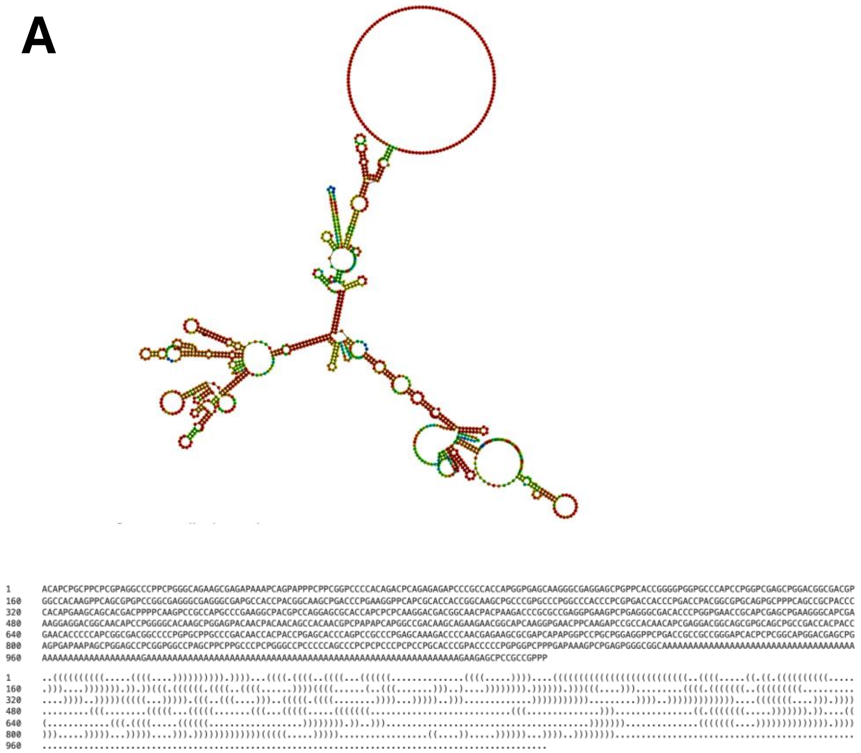

B

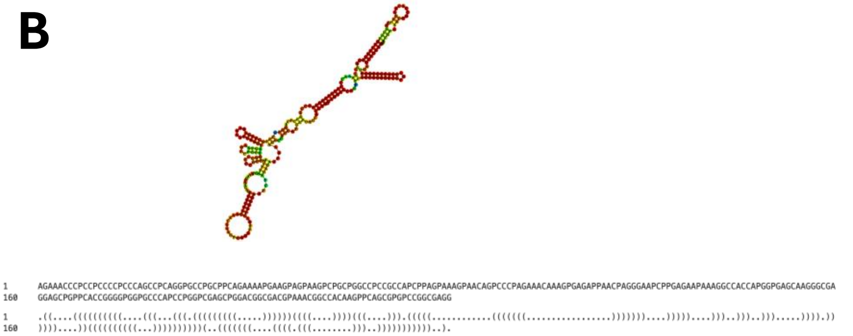

Figure S9. .RNAFold analysis for 5'-CD3e-EGFP, both for its full structure (A) and its first 100 coding sequnce base pairs (B)

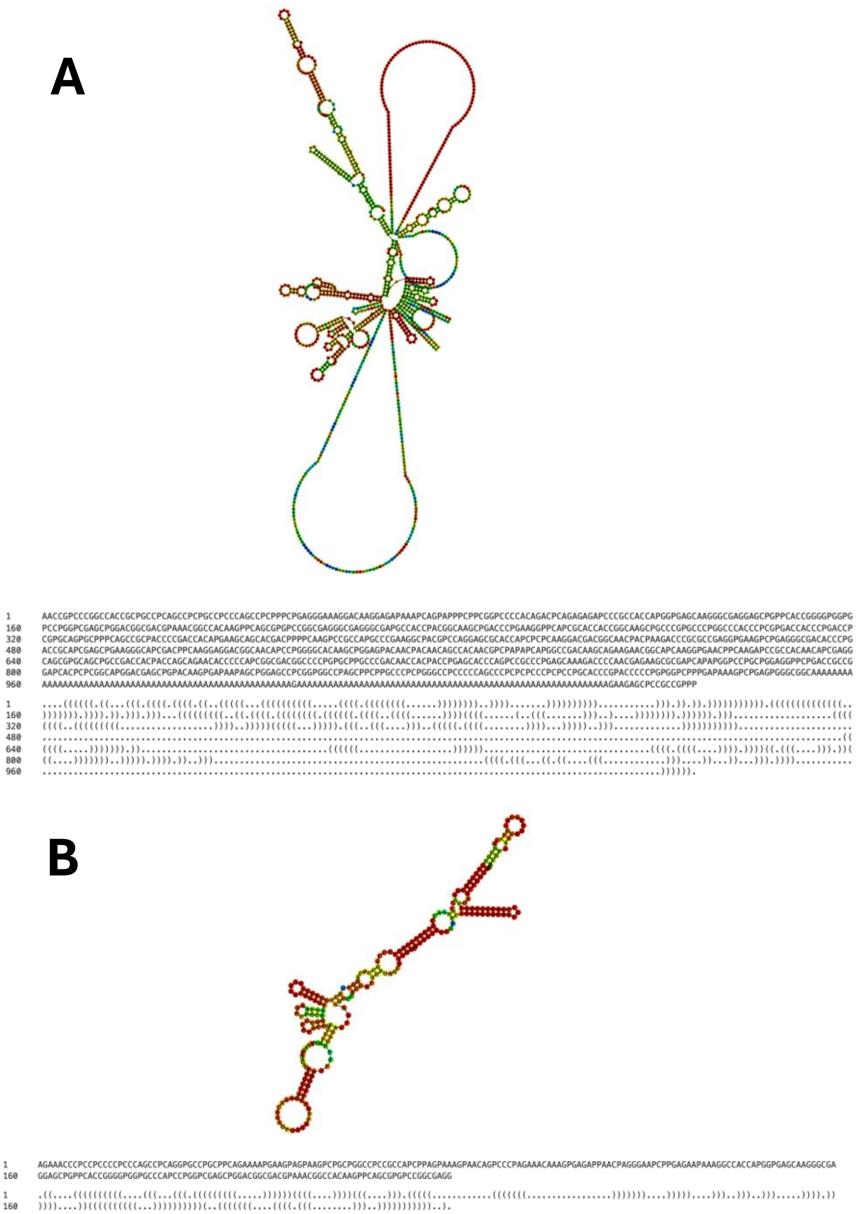

Figure S10. RNAFold analysis for 5'-CD247-EGFP, both for its full structure (A) and its first 100 coding sequnce base pairs (B)

A

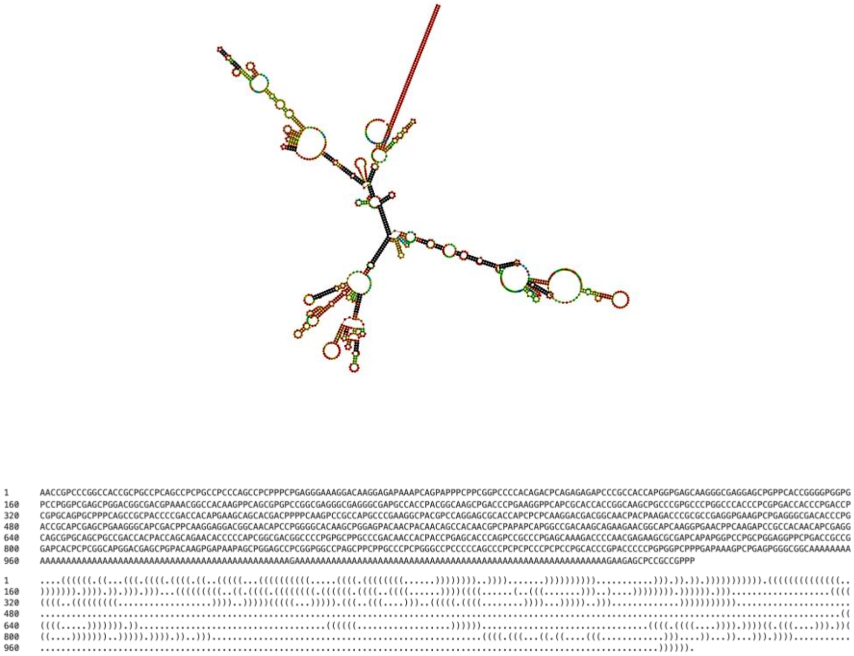

B

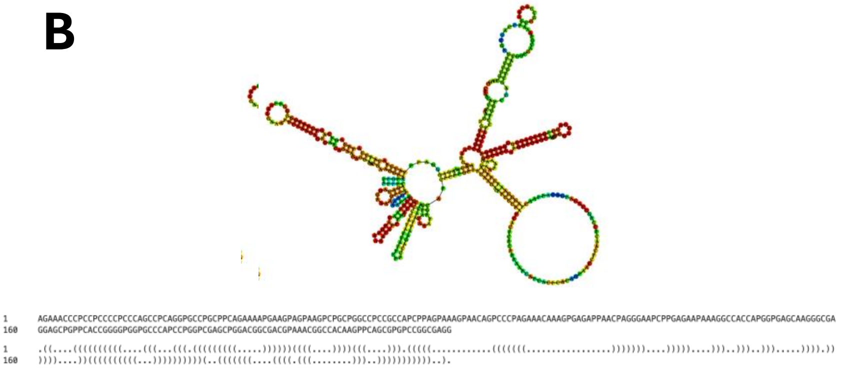

Il-2

Figure S11. RNAFold analysis for 5’-iL-2-EGFP, both for its full structure (A) and its first 100 coding sequnce base pairs (B)

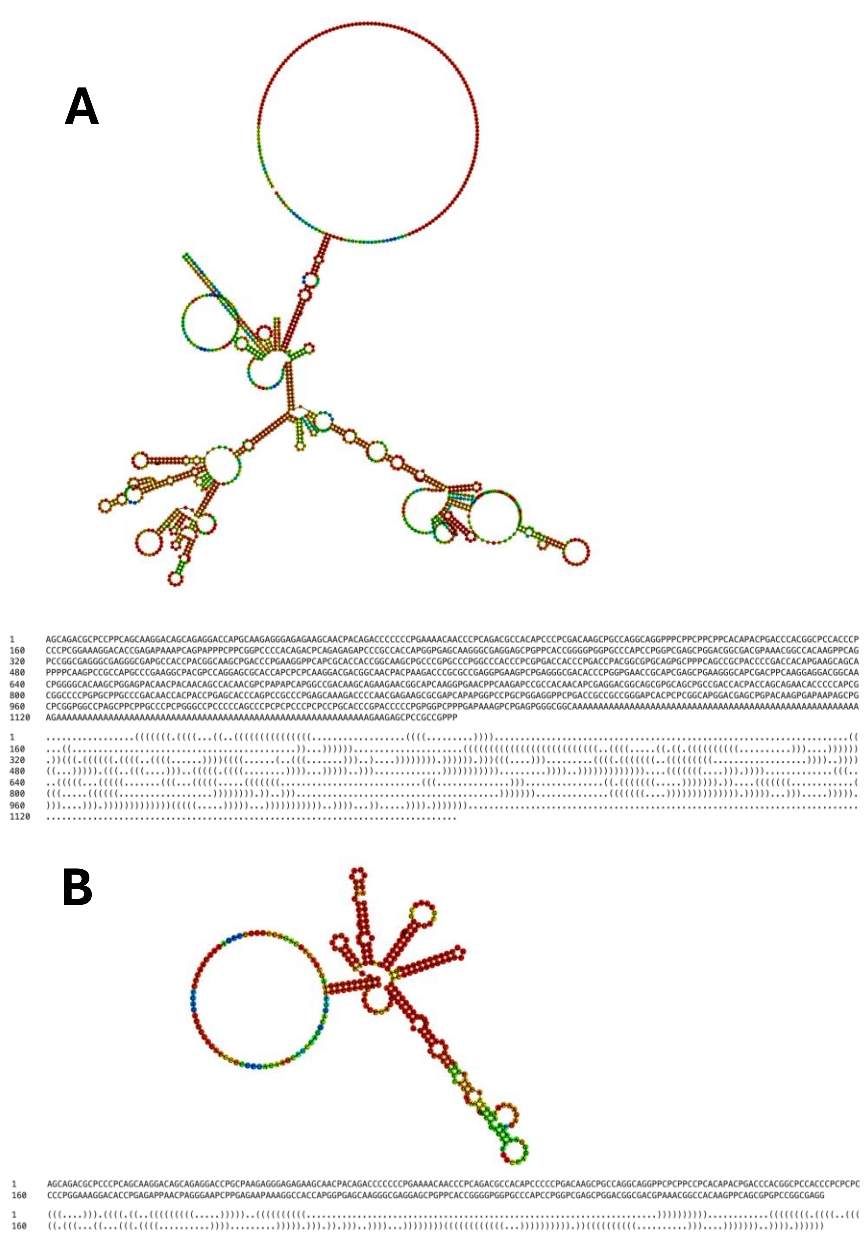

Figure S12. RNAFold analysis for 5’-TNF-EGFP, both for its full structure (A) and its first 100 coding sequnce base pairs (B)

A

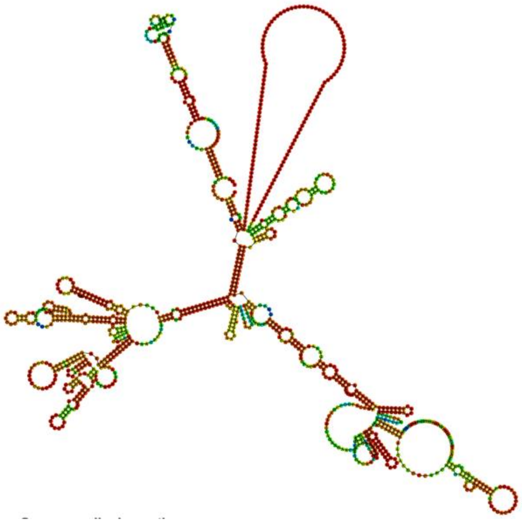

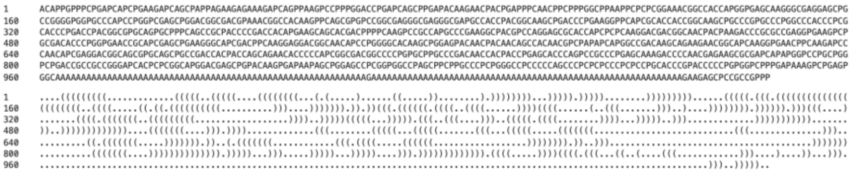

B

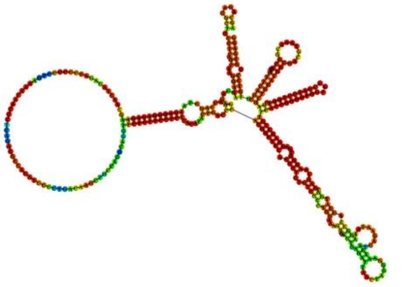

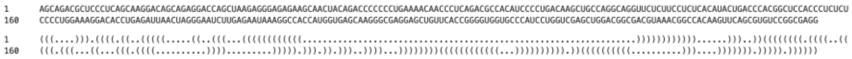

Figure S13. RNAFold analysis for 5'-IFNG-EGFP, both for its full structure (A) and its first 100 coding sequnce base pairs (B)

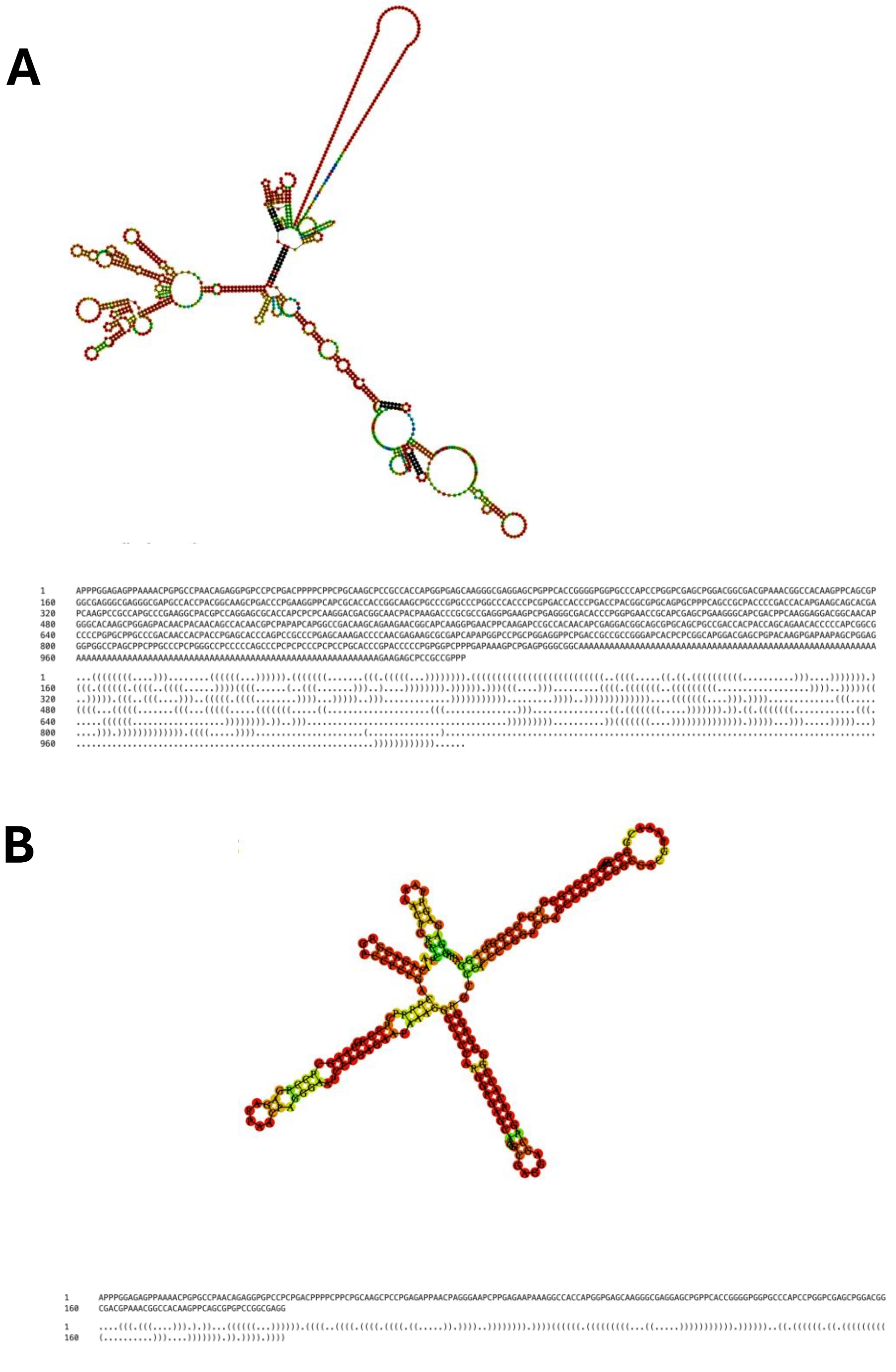

Figure S14. RNAFold analysis for 5'-TIM3F-EGFP, both for its full structure (A) and its first 100 coding sequnce base pairs (B)

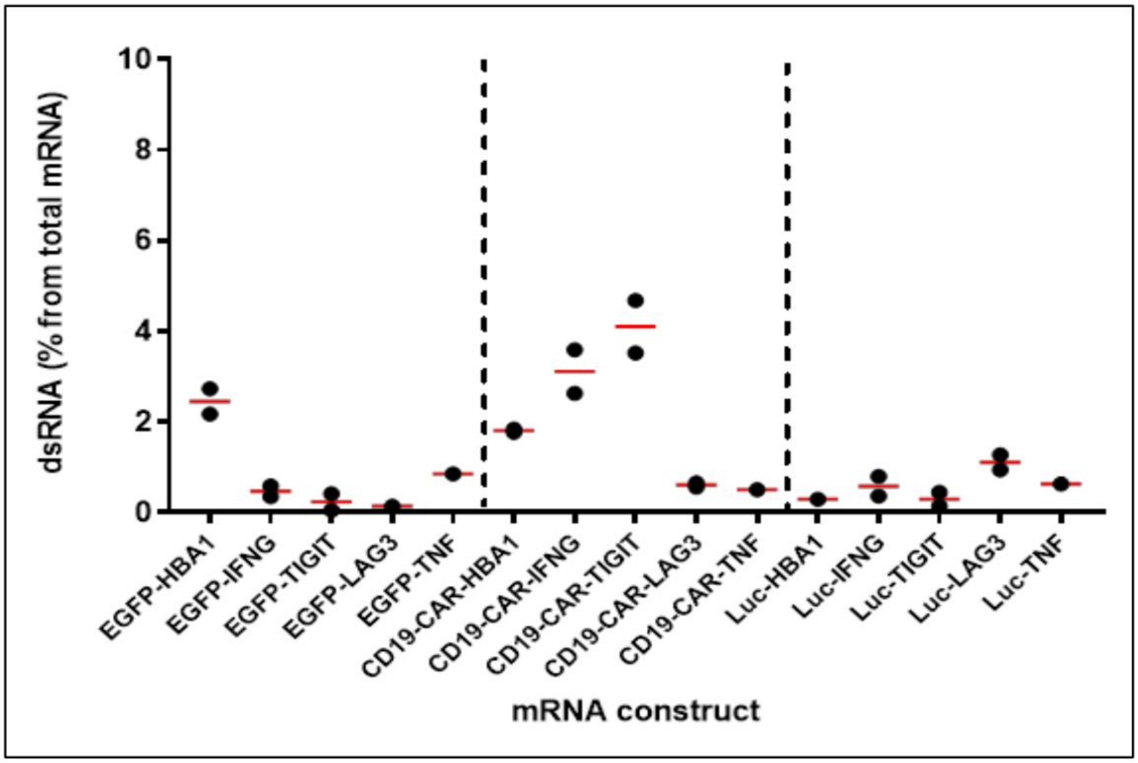

Figure S15. dsRNA content in IVT mRNA preparations. dsRNA was quantified using a J2-based anti-dsRNA assay (J2 dsRNA ELISA; Biorbyt) and expressed as a percentage of total mRNA Input for each construct. Points represent independent technical repeats; red bars indicate the mean. Values were interpolated from a positive-control standard curve using a sigmoidal 4-parameter logistic (4PL) fit
